# Cooperative Coverage Control for Heterogeneous AUVs Based on Control Barrier Functions and Consensus Theory

**DOI:** 10.3390/s26030822

**Published:** 2026-01-26

**Authors:** Fengxiang Mao, Dongsong Zhang, Liang Xu, Rui Wang

**Affiliations:** School of Big Data and Artificial Intelligence, Xinyang College, Xinyang 464000, China

**Keywords:** heterogeneous AUV swarms, cooperative coverage control, control barrier functions, consensus theory, quadratic programming

## Abstract

This paper addresses the problem of cooperative coverage control for heterogeneous Autonomous Underwater Vehicle (AUV) swarms operating in complex underwater environments. The objective is to achieve optimal coverage of a target region while simultaneously ensuring collision avoidance—both among AUVs and with static obstacles—and satisfying the inherent dynamic constraints of the AUVs. To this end, we propose a hierarchical control framework that fuses Control Barrier Functions (CBFs) with consensus theory. First, addressing the heterogeneity and limited sensing ranges of the AUVs, a cooperative coverage model based on a modified Voronoi partition is constructed. A nominal controller based on consensus theory is designed to balance the ratio of task workload to individual capability for each AUV. By minimizing a Lyapunov-like function via gradient descent, the swarm achieves self-organized optimal coverage. Second, to guarantee system safety, multiple safety constraints are designed for the AUV double-integrator dynamics, utilizing Zeroing Control Barrier Functions (ZCBFs) and High-Order Control Barrier Functions (HOCBFs). This approach unifies the handling of collision avoidance and velocity limitations. Finally, the nominal coverage controller and safety constraints are integrated into a Quadratic Programming (QP) formulation. This constitutes a safety-critical layer that modifies the control commands in a minimally invasive manner. Theoretical analysis demonstrates the stability of the framework, the forward invariance of the safe set, and the convergence of the coverage task. Simulation experiments verify the effectiveness and robustness of the proposed method in navigating obstacles and efficiently completing heterogeneous cooperative coverage tasks in complex environments.

## 1. Introduction

Covering over 70% of the Earth’s surface, the ocean harbors abundant natural resources and holds significant strategic importance for the global climate, economy, and security. As intelligent platforms capable of autonomous navigation and the execution of exploration and operational missions, Autonomous Underwater Vehicles (AUVs) have emerged as a pivotal technology for marine exploitation and utilization [1,2,3].

In recent years, driven by advancements in Multi-Agent System (MAS) theory, the deployment of multiple AUVs for cooperative missions has emerged as an inevitable trend in underwater operations [4,5]. The theoretical foundations of networked control in MASs have been extensively studied, providing rigorous frameworks for analyzing system stability and consensus [6,7]. Furthermore, concepts such as bearing rigidity have extended the capabilities of formation control and estimation beyond simple distance constraints [8]. In practical applications, ensuring collision avoidance while maintaining formation or tracking trajectories remains a critical challenge. Recent works have explored distributed formation tracking for second-order systems, emphasizing the importance of robust control protocols in dynamic environments [9]. Compared to a single AUV, an AUV swarm offers superior efficiency, enhanced robustness, and a more extensive operational range. In this context, cooperative coverage control represents a core application [10,11]. Its primary objective is to coordinate the AUVs to achieve an optimal distribution across the target region, thereby facilitating tasks such as seafloor mapping, hydrographic data collection, and search and rescue operations.

However, achieving efficient and safe cooperative coverage in practical underwater environments presents numerous challenges. First, AUV swarms are typically heterogeneous; that is, individual agents exhibit disparities in endurance, sensing capabilities, and data processing power [12]. Employing homogeneous control strategies in such scenarios can lead to the underutilization of capable AUVs and the overloading of less capable ones, thereby degrading overall system efficiency and operational endurance [13]. Consequently, it is imperative to design a heterogeneous cooperative coverage strategy capable of balancing task loads effectively. Second, the underwater environment is complex and unstructured, characterized by numerous known or unknown static obstacles (e.g., submerged reefs, shipwrecks, and subsea infrastructure). Furthermore, during mission execution, there is an inherent risk of inter-agent collisions [14]. Safety is the paramount prerequisite for AUV swarm operations. Traditional obstacle avoidance methods, such as Artificial Potential Fields (APFs), often suffer from local minima and struggle to provide rigorous safety guarantees [15]. Finally, AUVs are subject to complex nonlinear dynamics and physical constraints (e.g., velocity and acceleration saturation) [16], and their onboard sensors (e.g., sonar) typically possess limited sensing ranges [17]. Collectively, these factors significantly exacerbate the complexity of the control system design.

In the domain of AUV cooperative coverage, the Voronoi-based Lloyd’s algorithm stands as one of the most classical methods [18,19]. This approach operates by iteratively computing the centroids of Voronoi regions and steering agents toward these centroids, ultimately achieving optimal coverage of the region [20]. However, the conventional Lloyd’s algorithm is primarily tailored for homogeneous systems and fails to account for heterogeneity [21]. To address the challenge of heterogeneity, researchers have introduced workload-based consensus theory [22]. The core concept shifts from merely converging to region centroids to leveraging information exchange among neighbors; the objective is to drive the ratio of task workload to individual capability for each AUV toward a consensus value, thereby achieving load balancing in coverage control [23].

In terms of system safety assurance, Control Barrier Functions (CBFs) have garnered significant attention as an emerging technique [24]. By integrating CBFs with a nominal controller (e.g., a coverage controller) within a Quadratic Programming (QP) framework, it is possible to compute a control input online that prioritizes the satisfaction of safety constraints while executing the nominal task to the greatest extent possible [25]. This approach offers a powerful mathematical tool for addressing control problems in safety-critical systems.

To date, research applying CBFs to obstacle avoidance in multi-agent systems has yielded promising results; however, most existing studies predominantly concentrate on homogeneous systems or simple collision avoidance tasks. Research regarding the integration of the CBF safety framework with load-balanced coverage control for heterogeneous AUV swarms remains insufficient [26]. To address the aforementioned challenges, the aim of this study was to design a cooperative coverage control algorithm tailored for heterogeneous AUV swarms that provides rigorous safety guarantees. The main contributions of this paper are summarized as follows:

(a) A cooperative coverage model is proposed that explicitly accounts for both the heterogeneous capabilities of AUVs and their limited sensor sensing ranges. Based on a modified Voronoi partition, this model formalizes the coverage objective as the minimization of a global cost function that characterizes the variance of the task workload-to-capability ratio.
(b) A nominal controller based on gradient descent is designed specifically for the double-integrator dynamics of AUVs. The derivation of the gradient integrates consensus theory to drive the convergence of the workload-to-capability ratios, thereby achieving dynamic task load balancing at the system level.
(c) A unified safety-critical controller is constructed within a QP framework. By leveraging CBFs, this controller transforms inter-AUV collision avoidance, static obstacle avoidance, and velocity constraints into linear inequality constraints on the control input, ensuring rigorous system safety in complex environments.
(d) The effectiveness of the proposed control framework is rigorously proven. Utilizing Lyapunov stability theory and the invariance principle, it is demonstrated that the closed-loop system converges to the optimal equilibrium point of the coverage task subject to the satisfaction of all safety constraints.

The remainder of this paper is organized as follows. Section 2 presents the problem formulation and system modeling, covering preliminaries such as the CBF, the AUV dynamic model, and the modeling of the heterogeneous cooperative coverage problem. Section 3 details the design of the CBF-integrated coverage controller, including the derivation of the nominal controller gradient and the formalization of multiple safety constraints. Section 4 provides a system stability analysis of the proposed framework, encompassing proofs for both safety and coverage convergence. Section 5 validates the effectiveness of the proposed algorithm through numerical simulations. Finally, Section 6 concludes the paper and outlines directions for future research.

## 2. Problem Formulation and Modeling

This section serves to establish the theoretical and modeling foundations for the subsequent controller design. First, we introduce the preliminaries, including Control Barrier Functions—the cornerstone of the safety control strategy in this paper—and Voronoi theory for region partitioning. Next, the dynamic model of the AUV is established. Finally, we mathematically formulate the AUV cooperative coverage control problem by incorporating heterogeneity, limited sensing ranges, and safety constraints.

### 2.1. Preliminaries

#### 2.1.1. Control Barrier Functions

CBFs serve as a powerful tool for guaranteeing system safety. This is achieved by defining a safe set and ensuring that the system state consistently remains within the interior of this set.

Consider a nonlinear control-affine system described by

(1) $$\dot{x}=f\left(x\right)+g\left(x\right)u,$$

where $x\in R^{n}$ denotes the system state, and $u\in U\subset R^{m}$ represents the control input. The functions *f* and *g* are assumed to be locally Lipschitz-continuous [27].

**Definition 1**  (Safe Set and Forward Invariance)**.** *Consider a set C defined by a continuously differentiable function $h:R^{n}\to R$ as follows:*

(2) $$\left\{\begin{array}{cc} \; & C=\{x\in R^{n}:h\left(x\right)\geq 0\}\\ \; & \partial C=\{x\in R^{n}:h\left(x\right)=0\}\\ \; & Int\left(C\right)=\{x\in R^{n}:h\left(x\right)>0\} \end{array}\right.$$

The set *C* is referred to as the safe set. And *C* is said to be forward-invariant with respect to system (1) if, for every initial state $x(t_{0})\in C$, any solution x(t) to system (1) satisfies x(t)∈C for all $t\geq t_{0}$.

To ensure the forward invariance of *C*, the following definition is introduced:

**Definition 2**  (Extended Class K Function [28])**.** *A continuous function α:(−b,a)→(−∞,∞) (for some a,b>0) is called an extended class K function if it is strictly increasing and satisfies α(0)=0.*

**Definition 3**  (Zeroing Control Barrier Function, ZCBF)**.** *Given system (1) and the set C defined by (2), the function h(x) is called a Zeroing Control Barrier Function (ZCBF) if there exists an extended class K function α such that, for all x∈C, there exists a control input u∈U satisfying*

(3) $$\underset{u\in U}{\sup}\;\left[L_{f}h\left(x\right)+L_{g}h\left(x\right)u+\alpha \left(h\left(x\right)\right)\right]\geq 0,$$

*where* sup *denotes the supremum, and $L_{f}h\left(x\right)=\frac{\partial h}{\partial x}f\left(x\right)$ and $L_{g}h\left(x\right)=\frac{\partial h}{\partial x}g\left(x\right)$ are the Lie derivatives [28] of h(x) along f(x) and g(x), respectively.*

**Theorem 1**  (ZCBF and Safety [29]). *Given a ZCBF h(x), define the set of feasible controls $K_{cbf}\left(x\right)$ as*

(4) $$K_{cbf}\left(x\right)=\left\{u\in U|L_{f}h\left(x\right)+L_{g}h\left(x\right)u+\alpha \left(h\left(x\right)\right)\geq 0\right\}.$$

*For any initial state $x(t_{0})\in C$, if there exists a locally Lipschitz-continuous controller u(x) such that $u\left(x\right)\in K_{cbf}\left(x\right)$, then the system state x(t) will remain within C for all time, implying that C is forward-invariant.*

**Definition 4**  (Relative Degree)**.** *The relative degree r of a function $h:R^{n}\to R$ with respect to system (1) is defined as the minimum number of times h must be differentiated with respect to time for the control input u to explicitly appear in the derivative $\frac{d^{r}h}{dt^{r}}$.*

When the relative degree of h(x) satisfies r>1 (i.e., $L_{g}h\left(x\right)=0$), the control input *u* does not appear in inequality (4), rendering the ZCBF ineffective. In such cases, High-Order Control Barrier Functions (HOCBFs) are required.

**Definition 5**  (High-Order Control Barrier Function, HOCBF)**.** *For a function h(x) with a relative degree of r, a series of functions can be recursively defined as*

(5) $$\left\{\begin{array}{c} \psi_{0}\left(x\right)=h\left(x\right)\\ \psi_{1}\left(x\right)={\dot{\psi }}_{0}\left(x\right)+k_{1}\psi_{0}\left(x\right)\\ \vdots \\ \psi_{r-1}\left(x\right)={\dot{\psi }}_{r-2}\left(x\right)+k_{r-1}\psi_{r-2}\left(x\right) \end{array}\right.$$

*where $k_{1},\ldots ,k_{r-1}>0$ are constants. The function h(x) is referred to as a HOCBF if there exists an extended class K function α and a constant $k_{r}>0$ such that the set $C_{H}=\left\{x\in R^{n}|\psi_{0}\left(x\right)\geq 0,\ldots ,\psi_{r-1}\left(x\right)\geq 0\right\}$, defined by $\psi_{0},\ldots ,\psi_{r-1}$, is forward-invariant. This can be achieved by satisfying the following inequality:*

(6) $$L_{f}\psi_{r-1}\left(x\right)+L_{g}\psi_{r-1}\left(x\right)u+k_{r}\psi_{r-1}\left(x\right)\geq 0.$$

A controller *u* satisfying this inequality guarantees that the system state consistently remains within the safe set $C\subseteq C_{H}$.

#### 2.1.2. Control Lyapunov Functions

Control Lyapunov Functions (CLFs) serve as a fundamental tool for analyzing system stability and synthesizing stabilizing controllers. By extending Lyapunov stability theory to control systems, CLFs provide a means to identify a control input *u* that guarantees system stability.

**Definition 6**  (Control Lyapunov Function [30])**.** *For the control-affine system (1), a continuously differentiable function $V:R^{n}\to R$ is termed an (exponentially stabilizing) Control Lyapunov Function (CLF) if there exist positive constants $c_{1},c_{2},c_{3}$ such that for all $x\in R^{n}$:*

(7) $${c_{1}\left||x|\right|}^{2}\leq V\left(x\right)\leq c_{2}{\left||x|\right|}^{2},$$

(8) $$\underset{u\in U}{\inf}\;\left[L_{f}V\left(x\right)+L_{g}V\left(x\right)u+c_{3}V\left(x\right)\right]\leq 0.$$

Inequality (7) ensures the positive definiteness of V(x). Inequality (8) constitutes the core condition, indicating the existence of at least one control input *u* capable of rendering $\dot{V}\left(x,u\right)=L_{f}V\left(x\right)+L_{g}V\left(x\right)u$ negative definite or essentially causing V(x) to decay.

**Theorem 2**  (CLF and Controller). *Given a CLF V(x), any locally Lipschitz-continuous controller u(t) satisfying (8) stabilizes system (1) to the zero equilibrium state. In this work, the principles of CLFs are leveraged to design a nominal controller ${\hat{u}}_{i}$ that ensures the coverage cost function decays over time, thereby achieving convergence to the optimal coverage configuration.*

#### 2.1.3. Voronoi Region Partitioning

Voronoi partitioning stands as a classical and highly effective method for region decomposition in coverage control problems. Consider a swarm of *N* AUVs operating within a target region *Q*, with their positions denoted by $P=\{p_{1},p_{2},\ldots ,p_{N}\}$.

**Definition 7**  (Voronoi Partition [31])**.** *The Voronoi region $V_{i}$ associated with the i-th AUV $p_{i}$ is defined as the set of points within region Q whose distance to $p_{i}$*

(9) $$V_{i}\left(P\right)=\left\{q\in Q|f\left(p_{i},q\right)\leq f\left(p_{j},q\right),\forall j\in N\setminus \left\{i\right\}\right\},$$

*where $f(p_{i},q)$ typically represents the Euclidean distance between $p_{i}$ and q, i.e., $f\left(p_{i},q\right)=∥p_{i}-q∥$.*

**Lemma 1.**  *For a given cost function $H\left(P,W\right)=\sum_{i=1}^{N}\int_{W_{i}}f\left(p_{i},q\right)\phi \left(q\right)dq$, where ϕ(q) represents the scalar density function characterizing the information importance distribution (as defined in Section 2.3.1), under the condition that the AUV positions P are fixed, the optimal region partition $W=\{W_{1},\ldots ,W_{N}\}$ that minimizes H is precisely the Voronoi partition $V=\{V_{1},\ldots ,V_{N}\}$.*

### 2.2. AUV Dynamic Model

The complete six-degree-of-freedom (6-DOF) nonlinear dynamic model of an AUV is inherently complex [32]. In cooperative coverage control scenarios, AUV swarms typically execute planar missions at a constant depth or with fixed depth differentials. To reduce model complexity while retaining the essential inertial and velocity characteristics required for collision avoidance, the horizontal motion of the AUV is simplified to a double-integrator model [33]. Consider a swarm consisting of *N* AUVs, indexed by the set N={1,2,…,N}. The state of the *i*-th AUV is denoted as $x_{i}={\left[p_{i}^{T},v_{i}^{T}\right]}^{T}\in R^{4}$, where $p_{i}={\left[x_{i},y_{i}\right]}^{T}\in R^{2}$ represents its position in the horizontal plane, and $v_{i}={\left[{\dot{x}}_{i},{\dot{y}}_{i}\right]}^{T}\in R^{2}$ denotes its velocity. The dynamic model is expressed as follows:

(10) $$\left\{\begin{array}{c} {\dot{p}}_{i}=v_{i}\\ {\dot{v}}_{i}=u_{i} \end{array}\right.$$

where $u_{i}\in R^{2}$ serves as the control input, representing the horizontal acceleration of the AUV. This model aligns with the control-affine form described in (1), with the state defined as $x_{i}={\left[p_{i}^{T},v_{i}^{T}\right]}^{T}$ and the system dynamics given by

$$f\left(x_{i}\right)=\left[\begin{array}{c} v_{i}\\ 0_{2\times 1} \end{array}\right],\;g\left(x_{i}\right)=\left[\begin{array}{c} 0_{2\times 2}\\ I_{2\times 2} \end{array}\right]$$

Furthermore, the AUVs are subject to physical constraints, specifically limitations on the magnitudes of their velocity and acceleration:

(11) $$\left\{\begin{array}{c} \left|\right|v_{i}\left|\right|\leq v_{max}\\ \left|\right|u_{i}\left|\right|\leq u_{max} \end{array}\right.$$

where $v_{max}$ and $u_{max}$ denote the maximum velocity and maximum acceleration, respectively.

Although the double-integrator model simplifies complex hydrodynamics (e.g., drag and added mass), it serves as a high-level kinematic planner in this framework. The generated control input $u_{i}$ is treated as a reference acceleration, which is typically tracked by a low-level dynamic controller (e.g., PID or Sliding Mode) that handles the specific actuator dynamics and environmental disturbances in practical implementation.

### 2.3. Modeling of the Cooperative Coverage Control Problem

This section presents the mathematical formulation of the cooperative coverage problem for heterogeneous AUV swarms, explicitly accounting for limited sensor capabilities and safety constraints.

#### 2.3.1. Mission Space and Sensing Model

The mission space $Q\subset R^{2}$ for the AUV swarm is defined as a bounded convex region. The distribution of information importance within this region is characterized by a known density function $\phi :Q\to R_{\geq 0}$, where a larger value of ϕ(q) corresponds to a higher detection value or task priority at point *q*.

Each AUV is equipped with sonar sensors possessing limited sensing ranges. Consequently, the sensing range $C_{i}$ is modeled as a circular region centered at the AUV’s position $p_{i}$ with a radius $r_{i}$:

(12) $$C_{i}\left(p_{i}\right)=\{q\in Q|\left||q-p_{i}|\right|\leq r_{i},\forall i\in N\}.$$

The AUV swarm is heterogeneous, with each AUV possessing a distinct comprehensive mission capability denoted by $E_{i}\in R^{+}$. Here, $E_{i}$ is a scalar that comprehensively encapsulates factors such as sensor performance, data processing speed, and endurance. A larger value of $E_{i}$ indicates a stronger capability to execute missions.

Task Partitioning: Integrating Voronoi partitioning with sensor limitations, the region actually covered by an AUV is the intersection of its Voronoi cell and its sensing range, denoted as the sensing-limited Voronoi cell $V_{i}^{r_{i}}$:

(13) $$V_{i}^{r_{i}}\left(p\right)=V_{i}\left(p\right)\cap C_{i}\left(p_{i}\right).$$

This partitioning method may result in certain regions within *Q* (e.g., $V_{j}\setminus C_{j}$) remaining uncovered by any AUV, as these regions lie within the Voronoi cell of agent *j* but fall outside its sensing range.

To address this issue, a modified Voronoi partition $\Omega_{i}\left(p\right)$ is adopted. This approach reallocates the uncovered regions $S_{i}=\left({⋃}_{j\in N\setminus \{i\}}\left(V_{j}\setminus V_{j}^{r_{j}}\right)\right)\cap C_{i}$ to a neighbor *i* capable of sensing them. The final responsibility region $\Omega_{i}$ for AUV *i* is defined as

(14) $$\Omega_{i}=V_{i}^{r_{i}}\cup S_{i}\setminus \left(\underset{k\in N-\{i\}}{⋃}S_{ik}^{*}\right),$$

where $S_{ik}^{*}$ represents the portion of the overlap between $S_{i}$ and $S_{k}$ that is closer to *k*. This modified partition $\Omega_{i}$ ensures that all sensible regions are rationally allocated.

To compute the local Voronoi cell $V_{i}$, each AUV *i* only requires the relative positions of neighboring AUVs $j\in N_{i}$. Although the global domain boundary is assumed to be known a priori (as a stored map), the partition is computed in a fully distributed manner based on local interactions.

Workload Modeling: Based on this modified partition $\Omega_{i}\left(p\right)$, the task workload $T_{i}\left(p\right)$ of AUV *i* is defined as the integral of the information density within its responsibility region:

(15) $$T_{i}\left(p\right)=\int_{\Omega_{i}\left(p\right)}\phi \left(q\right)dq.$$

To balance the workload among the heterogeneous AUVs, the workload-to-capability ratio $R_{i}\left(p\right)$ is defined as

(16) $$R_{i}\left(p\right)=\frac{T_{i}\left(p\right)}{E_{i}}.$$

where $T_{i}\left(p\right)$ is the workload computed in Equation (15), and $E_{i}$ represents the heterogeneous mission capability coefficient of the *i*-th AUV.

To explicitly quantify the heterogeneous capability index $E_{i}$ from multi-dimensional physical attributes, we adopt a weighted linear aggregation method. The capability $E_{i}$ is computed as

$$E_{i}=\kappa \left(\omega_{v}\frac{v_{max,i}}{{\bar{v}}_{base}}+\omega_{s}\frac{R_{s,i}}{{\bar{R}}_{base}}+\omega_{e}\frac{T_{end,i}}{{\bar{T}}_{base}}\right),$$

where $v_{max,i}$, $R_{s,i}$, and $T_{end,i}$ represent the maximum velocity, sensing radius, and battery endurance of the *i*-th AUV, respectively. The denominators ${\bar{v}}_{base},{\bar{R}}_{base},{\bar{T}}_{base}$ are the maximum values of these attributes across the entire swarm, used for normalization to ensure all terms are dimensionless and scale-consistent. The coefficients $\omega_{v},\omega_{s},\omega_{e}\geq 0$ are user-defined weights satisfying $\omega_{v}+\omega_{s}+\omega_{e}=1$, and κ is a scaling factor.

The selection of these weighting coefficients dictates the sensitivity of the coverage strategy to specific attributes, allowing the framework to adapt to diverse mission requirements. For instance, in a time-critical rapid search mission, a higher value for $\omega_{v}$ can be assigned to prioritize agents with superior mobility. Conversely, for a detailed seabed mapping task requiring high-resolution data, $\omega_{s}$ would be dominant to leverage agents equipped with high-precision sonars. In the simulations presented in this paper, we assume a balanced configuration with equal weights to reflect a general-purpose scenario.

#### 2.3.2. Consensus and Cost Function Lemma

To achieve workload balancing among heterogeneous AUVs (i.e., driving the ratios $R_{i}=T_{i}/E_{i}$ toward a consensus), a global cost function is required such that its minimum corresponds to the state where all $R_{i}$ are consistent. The following lemma provides the mathematical basis for selecting the cost function $V_{c}=\sum \left(T_{i}^{2}/E_{i}\right)$.

**Lemma 2**  ([34]). *Consider n variables $\left\{\delta_{1},\delta_{2},\ldots ,\delta_{n}\right\}$ satisfying the constraint that their sum is a fixed constant, $\sum_{i=1}^{n}\delta_{i}=\Delta$. For a set of positive real numbers $\left\{\beta_{1},\beta_{2},\ldots ,\beta_{n}\right\}$, consider the objective function $V=\sum_{i=1}^{n}\frac{\delta_{i}^{2}}{\beta_{i}}$. The minimum value of V is given by*

(17) $$\min\left(V\right)=\min\left(\sum_{i=1}^{n}\frac{\delta_{i}^{2}}{\beta_{i}}\right)=\frac{\Delta^{2}}{\sum_{i=1}^{n}\beta_{i}}.$$

This minimum is attained if and only if the ratios of all variables $\frac{\delta_{i}}{\beta_{i}}$ are equal, i.e.,

(18) $$\frac{\delta_{1}}{\beta_{1}}=\frac{\delta_{2}}{\beta_{2}}=\ldots =\frac{\delta_{n}}{\beta_{n}}=\frac{\Delta }{\sum_{i=1}^{n}\beta_{i}}.$$

Application to Coverage: In the context of the coverage problem, let the variable $\delta_{i}$ correspond to the task workload $T_{i}$ of AUV *i*, and let the positive real number $\beta_{i}$ correspond to its mission capability $E_{i}$. Assuming that the total task workload $\sum T_{i}=\int_{Q}\phi \left(q\right)dq=\Delta$ is a constant, the constructed cost function $V_{c}\left(p\right)=\sum_{i=1}^{N}\frac{T_{i}{\left(p\right)}^{2}}{E_{i}}$ achieves its minimum if and only if $\frac{T_{i}}{E_{i}}=\frac{T_{j}}{E_{j}}$ (i.e., $R_{i}=R_{j}$). Consequently, designing a controller to minimize $V_{c}\left(p\right)$ effectively drives the system to realize heterogeneous load balancing.

#### 2.3.3. Control Objectives

The control objectives of this paper are classified into two aspects: the coverage objective and the safety objective.

(a) Coverage Objective: Through the autonomous movement of the AUV swarm, the positions *p* are dynamically adjusted such that the task workload-to-capability ratios $R_{i}$ tend toward consensus:
  (19) $$\lim_{t\to \infty }\;\left(R_{i}\left(p\left(t\right)\right)-R_{j}\left(p\left(t\right)\right)\right)=0,\;\forall i,j\in N.$$
  This realization of heterogeneous load balancing—where capable agents assume greater workloads—constitutes the core of the optimal cooperative coverage control.
(b) Safety Objective: System safety must be rigorously guaranteed throughout the execution of the coverage task. Leveraging CBF theory, the safety objective is formalized as maintaining the system state x(t) consistently within a safe set $C_{safe}$. The set $C_{safe}$ is defined as the intersection of multiple sub-safe sets:
  (20) $$C_{safe}=C_{agent}\cap C_{obs}\cap C_{vel},$$
  $C_{agent}$ (Inter-AUV Safety Set): To prevent collisions between AUV *i* and *j*, a safety function considering predictive braking is adopted. Let $d_{i}$ be the safety radius of AUV *i*, and the safe distance be $d_{safe,ij}=d_{i}+d_{j}$. The safety function $h_{ij}\left(x\right)\geq 0$ is defined as
  (21) $$h_{ij}\left(p,v\right)=2\sqrt{u_{max}(||\Delta p_{ij}\left|\right|-d_{safe,ij})}+\frac{\Delta p_{ij}^{T}\Delta v_{ij}}{||\Delta p_{ij}\left|\right|}\geq 0,\;\forall i\neq j,$$
  where $\Delta p_{ij}=p_{i}-p_{j}$ and $\Delta v_{ij}=v_{i}-v_{j}$. This function acts as a constraint primarily when $\Delta p_{ij}^{T}\Delta v_{ij}\leq 0$, i.e., when the AUVs are approaching each other.
  $C_{obs}$ (Obstacle Safety Set): To avoid collision between AUV *i* and a static obstacle *o* located at $p_{o}$ with radius $d_{o}$, the safe distance is defined as $d_{obs}=d_{i}+d_{o}$. The safety function $h_{io}\left(x\right)\geq 0$ is defined as
  (22) $$h_{io}\left(p\right)=\left|\right|p_{i}-p_{o}\left|\right|-d_{obs}\geq 0,\;\forall o\in O.$$
  This constitutes a constraint with a relative degree of 2, necessitating the use of HOCBFs.
  $C_{vel}$ (Velocity Limit Set): To ensure the velocity of AUV *i* does not exceed $v_{max}$, the safety function $h_{v,i}\left(x\right)\geq 0$ is defined as
  (23) $$h_{v,i}\left(v\right)=v_{max}^{2}-\left|\right|v_{i}|{|}^{2}\geq 0,\;\forall i\in N.$$
  This constitutes a constraint with a relative degree of 1.
  In practical AUV systems, non-negligible stopping distances arise due to inertia and drag. A conservative enlargement of the safety distance can be used to implicitly account for such effects. Qualitatively, the safety buffer $d_{safe}$ should be selected such that $d_{safe}>\frac{v_{max}^{2}}{2a_{brake}}+v_{max}\tau_{delay}$, where $a_{brake}$ is the maximum braking deceleration capability of the physical AUV subject to hydrodynamic drag, and $\tau_{delay}$ is the system response latency.

In summary, the core problem addressed in this paper is the design of a distributed control law $u_{i}$ such that, subject to the AUV dynamics (10) and physical constraints (11), the system state x(t) consistently satisfies the safety constraints (20) while simultaneously achieving the coverage objective (19).

## 3. Design of Coverage Control Method

**Figure 1:**
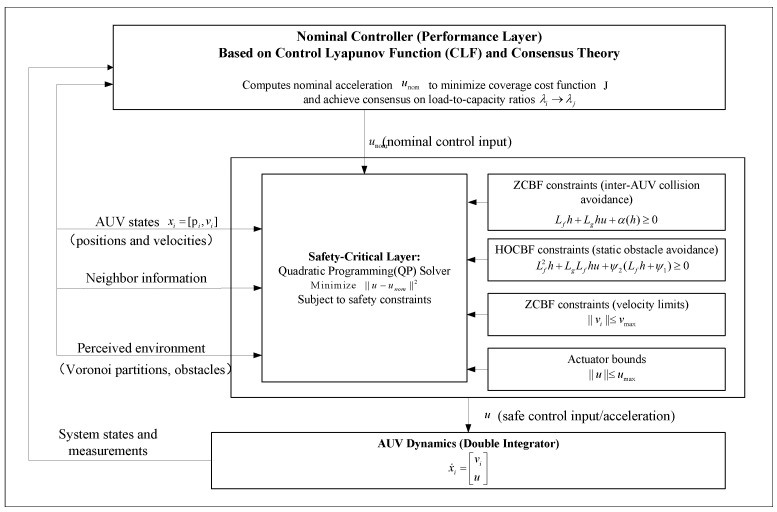
Hierarchical CBF-QP control framework for heterogeneous AUV cooperative coverage control.

This section details the design of a cooperative coverage controller for heterogeneous AUVs that explicitly incorporates safety constraints. A hierarchical control framework called CBF-QP is adopted. As illustrated in Figure 1, this framework comprises two primary components: a nominal controller and a safety layer.

Nominal Controller (${\hat{u}}_{i}$): Designed based on CLF concepts, this controller aims to achieve the coverage objective defined in Section 2.3.3—specifically, the consensus of the task workload-to-capability ratio $R_{i}$.

Safety Layer: Designed based on the CBF, this layer transforms all safety objectives defined in Section 2.3.3 (inter-AUV collision avoidance, obstacle avoidance, and velocity limits) into linear inequality constraints on the control input $u_{i}$.

The final control input $u_{i}^{*}$ is obtained by solving a QP problem. This QP formulation aims to minimize the deviation from the nominal controller ${\hat{u}}_{i}$ while strictly satisfying all CBF safety constraints:

(24) $$u_{i}^{*}=\arg\min_{u_{i}\in R^{2}}{\left||u_{i}-{\hat{u}}_{i}|\right|}^{2}\;s.t.\;\mathrm{CBF}\;safety\;constraints,$$

where $u_{i}^{*}$ denotes the final applied safety control input, and ${\hat{u}}_{i}$ represents the nominal control input designed to achieve coverage performance. The term ${∥\cdot ∥}^{2}$ is the squared Euclidean norm; minimizing this term implies that $u_{i}^{*}$ is the control action closest to ${\hat{u}}_{i}$ subject to the satisfaction of safety constraints.

### 3.1. Design of CLF-Based Nominal Controller ${\hat{u}}_{i}$

The primary objective of the nominal controller is to drive the system to converge to the coverage objective (19), specifically ensuring $R_{i}\to R_{j}$. This is realized by minimizing the global cost function $V_{c}\left(p\right)$.

#### 3.1.1. Control Lyapunov Function for Coverage Cost

A Control Lyapunov Function, denoted as $V_{clf}\left(x\right)$, is constructed comprising two components: the potential energy $V_{c}\left(p\right)$ characterizing the workload balance and the total kinetic energy of the system $V_{k}\left(v\right)$.

(25) $$V_{clf}\left(x\right)=V_{c}\left(p\right)+V_{k}\left(v\right)=\sum_{i=1}^{N}\frac{T_{i}{\left(p\right)}^{2}}{E_{i}}+\frac{1}{2}\sum_{i=1}^{N}v_{i}^{T}v_{i},$$

where $x={\left[p_{1}^{T},v_{1}^{T},\ldots ,p_{N}^{T},v_{N}^{T}\right]}^{T}$ represents the state vector of the entire system; $T_{i}\left(p\right)$ denotes the task workload of AUV *i*; $E_{i}$ represents the mission capability of AUV *i*; and $v_{i}$ is the velocity of AUV *i*. Based on Lemma 2, $V_{c}\left(p\right)$ attains its minimum if and only if the ratios $R_{i}=T_{i}/E_{i}$ are consistent across all agents.

The control objective is to design $u_{i}$ such that ${\dot{V}}_{clf}\leq 0$, thereby driving the system to converge to the minimum of $V_{clf}$. At this equilibrium, $v_{i}=0$, and the workload-to-capability ratios $R_{i}$ achieve consensus.

#### 3.1.2. Nominal Controller

First, compute the time derivative of the Control Lyapunov Function $V_{clf}\left(x\right)$:

(26) $${\dot{V}}_{clf}=\frac{d}{dt}V_{c}\left(p\right)+\frac{d}{dt}V_{k}\left(v\right)=\sum_{i=1}^{N}\left(\frac{\partial V_{c}}{\partial p_{i}}{\dot{p}}_{i}+\frac{\partial V_{k}}{\partial v_{i}}{\dot{v}}_{i}\right).$$

By substituting the AUV dynamic model (10) and noting that $\frac{\partial V_{k}}{\partial v_{i}}=v_{i}$, Equation (26) can be rewritten as

(27) $${\dot{V}}_{clf}=\sum_{i=1}^{N}\left({\left(\nabla_{p_{i}}V_{c}\right)}^{T}v_{i}+v_{i}^{T}u_{i}\right)=\sum_{i=1}^{N}v_{i}^{T}\left(\nabla_{p_{i}}V_{c}+u_{i}\right),$$

where $\nabla_{p_{i}}V_{c}$ denotes the gradient of the coverage cost with respect to the position of AUV *i*.

To render ${\dot{V}}_{clf}$ negative semi-definite, the nominal controller ${\hat{u}}_{i}$ is designed in the form of a proportional–derivative (PD) controller:

(28) $${\hat{u}}_{i}=-k_{p}\left(\nabla_{p_{i}}V_{c}\right)-k_{d}v_{i},$$

where $k_{p}>0$ and $k_{d}>0$ are control gains, corresponding to the proportional and derivative terms, respectively. For the sake of algebraic simplicity and without loss of generality, we set $k_{p}=1$ in the following derivation (noting that for any arbitrary $k_{p}>0$, the stability analysis follows identically by scaling the potential energy term in the Lyapunov candidate). Substituting Equation (28) into Equation (27) yields

(29) $${\dot{V}}_{clf}=\sum_{i=1}^{N}v_{i}^{T}\left(\nabla_{p_{i}}V_{c}+\left(-\nabla_{p_{i}}V_{c}-k_{d}v_{i}\right)\right)=-k_{d}\sum_{i=1}^{N}{\left||v_{i}|\right|}^{2}\leq 0.$$

This result demonstrates that the nominal controller ${\hat{u}}_{i}$ ensures the decay of $V_{clf}$, driving the system velocities $v_{i}\to 0$. Furthermore, according to LaSalle’s Invariance Principle [35], the system converges to the largest invariant set where ${\dot{V}}_{clf}=0$. This corresponds to the state where $v_{i}=0$ and $\nabla_{p_{i}}V_{c}=0$, which is precisely the coverage objective.

#### 3.1.3. Coverage Cost Gradient $\nabla_{p_{i}}V_{c}$

The core of the control law (28) lies in the computation of the gradient of $V_{c}\left(p\right)$ with respect to $p_{i}$, denoted as $\nabla_{p_{i}}V_{c}$.

(30) $$\nabla_{p_{i}}V_{c}=\frac{\partial V_{c}}{\partial p_{i}}=\frac{\partial }{\partial p_{i}}\sum_{k=1}^{N}\frac{T_{k}{\left(p\right)}^{2}}{E_{k}}=\sum_{k=1}^{N}\frac{2T_{k}\left(p\right)}{E_{k}}\frac{\partial T_{k}\left(p\right)}{\partial p_{i}}.$$

Based on Equation (16), where $R_{k}\left(p\right)=T_{k}\left(p\right)/E_{k}$, the above expression can be rewritten as

(31) $$\nabla_{p_{i}}V_{c}=2\sum_{k=1}^{N}R_{k}\left(p\right)\frac{\partial T_{k}\left(p\right)}{\partial p_{i}}.$$

Since the task workload $T_{k}\left(p\right)=\int_{\Omega_{k}\left(p\right)}\phi \left(q\right)dq$ for AUV *k* is defined over the responsibility region $\Omega_{k}\left(p\right)$, the boundary of $\Omega_{k}\left(p\right)$ depends solely on the position $p_{k}$ and the positions of its neighbors $p_{j}$ (where $j\in N_{i}$, and $N_{i}$ denotes the set of neighbors of *i*). Consequently, for any $k\notin \left\{i\right\}\cup N_{i}$, the derivative vanishes, i.e., $\frac{\partial T_{k}}{\partial p_{i}}=0$. Equation (31) can thus be simplified to

(32) $$\nabla_{p_{i}}V_{c}=2R_{i}\frac{\partial T_{i}}{\partial p_{i}}+2\sum_{j\in N_{i}}R_{j}\frac{\partial T_{j}}{\partial p_{i}}.$$

To compute $\frac{\partial T_{k}}{\partial p_{i}}$, the Leibniz integral rule [36] is applied. Since the density function ϕ(q) is independent of the AUV position $p_{i}$ (i.e., $\frac{\partial \phi (q)}{\partial p_{i}}=0$), only the boundary terms need to be considered:

(33) $$\frac{\partial T_{k}\left(p\right)}{\partial p_{i}}=\frac{\partial }{\partial p_{i}}\int_{\Omega_{k}\left(p\right)}\phi \left(q\right)dq=\int_{\partial \Omega_{k}}\phi \left(q\right)n_{k}^{T}\left(q\right)\frac{\partial q}{\partial p_{i}}ds,$$

where $\partial \Omega_{k}$ denotes the boundary of $\Omega_{k}$, and $n_{k}\left(q\right)$ represents the outward unit normal vector at point *q* on the boundary.

(a) Case k=i: The boundary $\partial \Omega_{i}$ consists of two parts: the boundary $\partial \Omega_{ij}$ shared with neighbor *j*, and the sensing boundary $\partial \Omega_{i}^{s}$ (the arc where $∥q-p_{i}∥=r_{i}$). Thus,
  (34) $$\frac{\partial T_{i}}{\partial p_{i}}=\sum_{j\in N_{i}}\int_{\partial \Omega_{ij}}\phi \left(q\right)n_{i}^{T}\left(q\right)\frac{\partial q}{\partial p_{i}}ds+\int_{\partial \Omega_{i}^{s}}\phi \left(q\right)n_{i}^{T}\left(q\right)\frac{\partial q}{\partial p_{i}}ds.$$
(b) Case k=j ($j\in N_{i}$): For a neighbor *j*, only the portion of the boundary $\partial \Omega_{j}$ that is shared with *i* (i.e., $\partial \Omega_{ji}=\partial \Omega_{ij}$) depends on $p_{i}$. Thus,
  (35) $$\frac{\partial T_{j}}{\partial p_{i}}=\int_{\partial \Omega_{ji}}\phi \left(q\right)n_{j}^{T}\left(q\right)\frac{\partial q}{\partial p_{i}}ds.$$

Note that on the shared boundary, the normal vectors are opposite, i.e., $n_{j}\left(q\right)=-n_{i}\left(q\right)$. Substituting (34) and (35) into the gradient formula (32) yields

(36) $$\begin{array}{cc} \nabla_{p_{i}}V_{c} & =2R_{i}\left(\sum_{j\in N_{i}}\int_{\partial \Omega_{ij}}\phi \left(q\right)n_{i}^{T}\left(q\right)\frac{\partial q}{\partial p_{i}}ds+\int_{\partial \Omega_{i}^{s}}\phi \left(q\right)n_{i}^{T}\left(q\right)\frac{\partial q}{\partial p_{i}}ds\right)\\ & \;+2\sum_{j\in N_{i}}R_{i}\left(\int_{\partial \Omega_{ij}}\phi \left(q\right)\left(-n_{i}^{T}\left(q\right)\right)\frac{\partial q}{\partial p_{i}}ds\right), \end{array}$$

where $\nabla_{p_{i}}V_{c}$ is the gradient of the global cost function $V_{c}$ with respect to the position $p_{i}$ of the AUV*i*. $R_{i}$ and $R_{j}$ are the task load-capability ratios of AUV*i* and its neighbor *j*, respectively, and $R_{i}=T_{i}/E_{i}$. $N_{i}$ is the neighbor set of AUV*i*. $\partial \Omega_{ij}$ is the shared Voronoi boundary between AUV*i* and *j*. $\partial \Omega_{i}^{s}$ is the sensing boundary of AUV*i* that is not shared with any neighbors (i.e., the part of $||q-p_{i}\left|\right|=r_{i}$). ϕ(q) is the task density at point *q*. $n_{i}^{T}\left(q\right)$ is the transpose of the outward normal vector of the boundary $n_{i}\left(q\right)$ at point *q*. $\frac{\partial q}{\partial p_{i}}$ is the sensitivity of the points *q* on the boundary to the movement of $p_{i}$. ds denotes the line integral along the boundary.

By rearranging the terms associated with the shared boundaries, the following is obtained:

(37) $$\nabla_{p_{i}}V_{c}=2\sum_{j\in N_{i}}\left(R_{i}-R_{j}\right)\left[\int_{\partial \Omega_{ij}}\phi \left(q\right)n_{i}^{T}\left(q\right)\frac{\partial q}{\partial p_{i}}ds\right]+2R_{i}\left[\int_{\partial \Omega_{i}^{s}}\phi \left(q\right)n_{i}^{T}\left(q\right)\frac{\partial q}{\partial p_{i}}ds\right].$$

The gradient $\nabla_{p_{i}}V_{c}$ possesses distinct physical interpretations:

(a) Consensus Term (First Term): This term represents the weighted sum of the differences between $R_{i}$ and $R_{j}$. The term $\left(R_{i}-R_{j}\right)$ indicates that if the workload ratio of AUV *i* exceeds that of its neighbor *j*, the gradient generates a force driving $p_{i}$ to move—typically away from *j*—to reduce $T_{i}$ and increase $T_{j}$, thereby driving $R_{i}$ and $R_{j}$ toward consensus.
(b) (Second Term): This term reflects the task density along the sensing boundary $\partial \Omega_{i}^{s}$ of AUV *i*. If the density ϕ(q) at the boundary is high, this term generates a force pushing AUV *i* toward the high-density region to expand coverage.

Consequently, the nominal controller ${\hat{u}}_{i}$ in Equation (28) is explicitly expressed as

(38) $${\hat{u}}_{i}=-k_{p}\left(2\sum_{j\in N_{i}}\left(R_{i}-R_{j}\right)F_{ij}\left(p\right)+2R_{i}F_{is}\left(p_{i}\right)\right)-k_{d}v_{i},$$

where $F_{ij}\left(p\right)$ and $F_{is}\left(p_{i}\right)$ represent the two boundary integral vectors defined in (37), respectively.

To enable the distributed computation of Equation (38) and the Voronoi partition, each AUV *i* is required to broadcast a local state packet to its neighbors $j\in N_{i}$ at each control cycle. The exchanged information set includes $\left\{p_{i},v_{i},E_{i},T_{i}\right\}$, where $p_{i},v_{i}$ are for collision avoidance and Voronoi partitioning, and $E_{i},T_{i}$ (or the ratio $R_{i}=T_{i}/E_{i}$) are for the consensus controller.

### 3.2. Design of Safety Constraints

(1) Constraint 1: Inter-AUV Collision Avoidance

Utilizing the Zeroing Control Barrier Function $h_{ij}\left(x\right)$ defined in (21),

(39) $$h_{ij}\left(p,v\right)=2\sqrt{u_{max}(||\Delta p_{ij}\left|\right|-d_{safe,ij})}+\frac{\Delta p_{ij}^{T}\Delta v_{ij}}{||\Delta p_{ij}\left|\right|}\geq 0,$$

where $h_{ij}\left(p,v\right)$ is the safety function between AUV *i* and *j*. $\Delta p_{ij}=p_{i}-p_{j}$ denotes the relative position, and $\Delta v_{ij}=v_{i}-v_{j}$ denotes the relative velocity. $u_{max}$ is the maximum acceleration, and $d_{safe,ij}$ is the minimum safety distance.

This function possesses a relative degree of r=1. According to Theorem 1, the safety constraint is formulated as ${\dot{h}}_{ij}+\lambda_{1}h_{ij}\geq 0$, where $\lambda_{1}>0$ is a tunable parameter. The complete form of ${\dot{h}}_{ij}$ is given by

(40) $${\dot{h}}_{ij}=L_{f}h_{ij}+L_{g}h_{ij}\Delta u_{ij}=L_{f}h_{ij}+L_{g}h_{ij}\left(u_{i}-u_{j}\right),$$

where $\Delta u_{ij}=u_{i}-u_{j}$. The Lie derivatives are defined as $L_{f}h_{ij}={\left(\nabla_{p}h_{ij}\right)}^{T}\dot{p}+{\left(\nabla_{v}h_{ij}\right)}^{T}f\left(x\right)$ and $L_{g}h_{ij}={\left(\nabla_{v}h_{ij}\right)}^{T}g\left(x\right)=\frac{\Delta p_{ij}^{T}}{∥\Delta p_{ij}∥}$.

Consequently, the safety constraint transforms into

(41) $$L_{f}h_{ij}+\frac{\Delta p_{ij}^{T}}{||\Delta p_{ij}\left|\right|}\left(u_{i}-u_{j}\right)+\lambda_{1}h_{ij}\geq 0.$$

**Remark 1.**  *(Parameter Tuning for $\lambda_{1}$). The parameter $\lambda_{1}>0$ in the ZCBF constraint serves as a stiffness gain that dictates how aggressively the AUV is allowed to approach the safety boundary. A larger $\lambda_{1}$ permits the system to operate closer to the neighbor before intervening, resulting in less conservative behavior but demanding higher control authority (acceleration) to brake or turn sharply. Conversely, a smaller $\lambda_{1}$ enforces a more conservative safety margin, triggering avoidance maneuvers earlier but with smoother control inputs. In practical implementation, $\lambda_{1}$ should be tuned empirically to maximize maneuverability while strictly ensuring that the required safety control input does not exceed the actuator’s physical saturation limit.*

To decouple the control inputs $u_{i}$ and $u_{j}$ within the QP formulation, a symmetrical Responsibility Allocation strategy is adopted. This distributes the safety constraint burden equally between agents *i* and *j*:

(42) $$\frac{\Delta p_{ij}^{T}}{||\Delta p_{ij}\left|\right|}u_{i}\geq -\frac{1}{2}\left(L_{f}h_{ij}+\lambda_{1}h_{ij}\right).$$

It is noted that the current formulation primarily addresses static obstacles. For dynamic non-cooperative obstacles, the constraint (42) can be adapted by estimating the obstacle’s velocity vector and treating it as a time-varying boundary constraint.

The inter-AUV collision avoidance constraint is thus formally defined as a linear inequality:

(43) $$A_{ij}u_{i}\geq b_{ij}\left(x\right),$$

where the coefficient matrix $A_{ij}$ and the boundary vector $b_{ij}\left(x\right)$ are defined as

$$A_{ij}=\frac{\Delta p_{ij}^{T}}{∥\Delta p_{ij}∥},\;b_{ij}\left(x\right)=-\frac{1}{2}\left(L_{f}h_{ij}\left(p,v\right)+\lambda_{1}h_{ij}\left(p,v\right)\right)$$

and the Lie derivative $L_{f}h_{ij}$ is explicitly calculated as

$$L_{f}h_{ij}=\frac{u_{max}\Delta p_{ij}^{T}\Delta v_{ij}}{\sqrt{u_{max}(∥\Delta p_{ij}∥-d_{safe,ij})}∥\Delta p_{ij}∥}+\frac{∥\Delta v_{ij}{∥}^{2}}{∥\Delta p_{ij}∥}-\frac{{\left(\Delta p_{ij}^{T}\Delta v_{ij}\right)}^{2}}{∥\Delta p_{ij}{∥}^{3}}$$

(2) Constraint 2: Static Obstacle Avoidance

Using the function $h_{io}\left(p\right)=∥p_{i}-p_{o}∥-d_{obs}\geq 0$ defined in Equation (22), this function has a relative degree of r=2 with respect to $u_{i}$ (since ${\dot{h}}_{io}$ depends only on $v_{i}$, and ${\ddot{h}}_{io}$ depends on $u_{i}$). Based on the HOCBF definition in Definition 5, define $\psi_{0}\left(x\right)=h_{io}\left(x\right)$ and $\psi_{1}\left(x\right)$ as

$$\psi_{1}\left(x\right)={\dot{\psi }}_{0}\left(x\right)+k_{1}\psi_{0}\left(x\right)=L_{f}h_{io}\left(x\right)+k_{1}h_{io}\left(x\right)$$

The HOCBF safety constraint is then given by ${\dot{\psi }}_{1}\left(x\right)+k_{2}\psi_{1}\left(x\right)\geq 0$, where $k_{1},k_{2}>0$. Then,

(44) $${\dot{\psi }}_{1}=\frac{\partial \psi_{1}}{\partial x}\dot{x}=L_{f}\psi_{1}+L_{g}\psi_{1}u_{i}.$$

Expanding $L_{f}\psi_{1}$ and $L_{g}\psi_{1}$ yields

(45) $$\left\{\begin{array}{c} L_{f}\psi_{1}=L_{f}\left(L_{f}h_{io}+k_{1}h_{io}\right)=L_{f}^{2}h_{io}+k_{1}L_{f}h_{io}\\ L_{g}\psi_{1}=L_{g}\left(L_{f}h_{io}+k_{1}h_{io}\right)=L_{g}L_{f}h_{io}+k_{1}L_{g}h_{io} \end{array}\right.$$

Since $h_{io}$ is solely a function of *p*, $L_{g}h_{io}=\frac{\partial h_{io}}{\partial v_{i}}=0$. Substituting (45) into (44), the constraint becomes

(46) $$\left(L_{f}^{2}h_{io}+k_{1}L_{f}h_{io}\right)+\left(L_{g}L_{f}h_{io}\right)u_{i}+k_{2}\left(L_{f}h_{io}+k_{1}h_{io}\right)\geq 0,$$

where the terms are explicitly calculated as

$$L_{f}h_{io}={\left(\nabla_{p_{i}}h_{io}\right)}^{T}v_{i}=\frac{{\left(p_{i}-p_{o}\right)}^{T}v_{i}}{\left|\right|p_{i}-p_{o}\left|\right|},$$

$$A_{io}=L_{g}L_{f}h_{io}={\left(\nabla_{v_{i}}\left(L_{f}h_{io}\right)\right)}^{T}=\frac{{\left(p_{i}-p_{o}\right)}^{T}}{\left|\right|p_{i}-p_{o}\left|\right|},$$

$$L_{f}^{2}h_{io}={\left(\nabla_{p_{i}}\left(L_{f}h_{io}\right)\right)}^{T}v_{i}=\frac{\left|\right|v_{i}|{|}^{2}}{\left|\right|p_{i}-p_{o}\left|\right|}-\frac{{\left({\left(p_{i}-p_{o}\right)}^{T}v_{i}\right)}^{2}}{\left|\right|p_{i}-p_{o}|{|}^{3}},$$

$$b_{io}\left(x\right)=-\left(L_{f}^{2}h_{io}+\left(k_{1}+k_{2}\right)L_{f}h_{io}+k_{1}k_{2}h_{io}\right).$$

(3) Constraint 3: Velocity Limit

Using the function $h_{v,i}\left(v\right)=v_{max}^{2}-{∥v_{i}∥}^{2}\geq 0$ defined in Equation (22), this function has a relative degree of r=1. The ZCBF constraint is given by ${\dot{h}}_{v,i}+\lambda_{3}h_{v,i}\geq 0$. Thus,

(47) $${\dot{h}}_{v,i}=L_{f}h_{v,i}+L_{g}h_{v,i}u_{i}.$$

Since $h_{v,i}$ depends solely on *v*, $L_{f}h_{v,i}=\frac{\partial h_{v,i}}{\partial p_{i}}v_{i}=0$, and $L_{g}h_{v,i}=\frac{\partial h_{v,i}}{\partial v_{i}}=-2v_{i}^{T}$. The constraint transforms into

(48) $$-2v_{i}^{T}u_{i}+\lambda_{3}\left(v_{max}^{2}-\left|\right|v_{i}|{|}^{2}\right)\geq 0.$$

The velocity limit constraint is formally defined as a linear inequality:

(49) $$A_{v,i}u_{i}\leq b_{v,i}\left(x\right),$$

where $A_{v,i}=2v_{i}^{T}$ and $b_{v,i}\left(x\right)=\lambda_{3}(v_{max}^{2}-∥v_{i}{∥}^{2})$.

The nominal controller ${\hat{u}}_{i}$ from (38) and all safety constraints (43), (47), and (50) are combined into a single Quadratic Programming problem. Simultaneously, the acceleration limits from Equation (11) are incorporated as boundary constraints for the QP.

To guarantee the feasibility of the optimization problem under all conditions and to explicitly define the constraint activation logic, we reformulate the QP with a relaxation (slack) variable δ. The revised optimization problem for each AUV *i* is formulated as

(50) $$\begin{array}{cc} u_{i}^{*},\delta^{*}=\arg\min_{u_{i},\delta }\; & \left|\right|u_{i}-{\hat{u}}_{i}{\left|\right|}^{2}+p\cdot \delta^{2}\\ s.t.\; & A_{ij}u_{i}\geq b_{ij}\left(x\right)-\delta ,\;\forall j\in N_{i}^{active}\\ \; & A_{io}u_{i}\geq b_{io}\left(x\right)-\delta ,\;\forall o\in O_{i}^{active}\\ \; & A_{v,i}u_{i}\leq b_{v,i}\left(x\right)\\ \; & \left|\right|u_{i}\left|\right|\leq u_{max} \end{array}$$

where p>0 is a sufficiently large penalty coefficient prioritizing safety, and $u_{max}$ represents the physical actuator saturation limit. Here, $u_{i}^{*}$ denotes the optimal control input, and $\delta^{*}$ represents the optimal value of the slack variable, which quantifies the minimum necessary violation of safety constraints. The scalar slack variable δ≥0 transforms the original hard safety constraints into soft constraints. This formulation provides a sufficient condition for feasibility: For any system state *x*, there implies the existence of a sufficiently large δ such that the feasible set of (50) is non-empty. Consequently, in extreme scenarios where strictly satisfying all safety constraints is physically impossible (e.g., deadlock), the solver yields a non-zero $\delta^{*}$ as an automatic relaxation strategy, finding a solution that minimizes the safety violation to maintain control continuity rather than failing.

The active sets are determined by the AUV’s local sensing capability. The active neighbor set is defined as $N_{i}^{active}=\{j\in N|||p_{i}-p_{j}\left|\right|\leq R_{s}\}$ and the active obstacle set as $O_{i}^{active}=\{o\in O|||p_{i}-p_{o}\left|\right|\leq R_{s}\}$, where $R_{s}$ is the AUV’s maximum sensing radius defined in Equation (12). To prevent high-frequency chattering when obstacles are near the sensing boundary, a hysteresis mechanism is applied: a constraint is deactivated only when the distance exceeds $R_{s}+\epsilon_{hys}$, where $\epsilon_{hys}>0$ is a small user-defined distance buffer constant (e.g., $\epsilon_{hys}=0.5\;m$).

This QP formulation constitutes a convex optimization problem, which allows for efficient online solution. The resulting $u_{i}^{*}$ represents the final control command applied to AUV *i*, which guarantees safety while maximally approximating the nominal control objective for coverage.

## 4. System Stability Analysis

This section aims to provide a rigorous mathematical proof of the two fundamental properties characterizing the CBF-QP control framework (51) proposed in Section 3: system safety and coverage convergence.

(1) Safety Analysis: Under the effect of the proposed QP controller $u_{i}^{*}$, the system state x(t) remains invariant within the safety set $C_{safe}$ defined in Equation (20). Specifically, the AUVs are guaranteed to avoid collisions with other agents and obstacles and to strictly adhere to velocity limits at all times.

(2) Convergence Analysis: Subject to the safety guarantees, the system state x(t) asymptotically converges to a stable equilibrium. At this equilibrium, the AUV velocities vanish (i.e., $v_{i}=0$), and the heterogeneous coverage cost function $V_{c}\left(p\right)$ attains a local minimum.

### 4.1. Safety Analysis

System safety is realized by ensuring the forward invariance of the safety set $C_{safe}$.

**Theorem 3.**  *(System Safety) For the AUV swarm system, consider the safety set $C_{safe}$ defined in Equation (20). If the initial system state $x\left(0\right)\in C_{safe}$, then the closed-loop system formed by the QP controller $u_{i}^{*}$ guarantees that the safe set $C_{safe}$ is forward-invariant, provided that the optimal slack variable satisfies $\delta^{*}\left(t\right)=0$ for all t≥0. In extreme scenarios where strictly satisfying all constraints is physically impossible ($\delta^{*}>0$), the controller minimizes the violation of the safety boundary to maintain operational continuity.*

**Proof of Theorem 3.**  According to Equation (20), the safety set $C_{safe}$ is the intersection of three subsets: $C_{safe}=C_{agent}\cap C_{obs}\cap C_{vel}$. By set theory, to prove that $C_{safe}$ is forward-invariant, it suffices to prove that each of the three sets—$C_{agent}$, $C_{obs}$, and $C_{vel}$—is individually forward-invariant.

(a) Forward Invariance of $C_{agent}$ (Inter-AUV Collision Avoidance):
  The set $C_{agent}$ is characterized by the safety function $h_{ij}\left(x\right)\geq 0$, where
  $$h_{ij}\left(p,v\right)=2\sqrt{u_{max}(||\Delta p_{ij}\left|\right|-d_{safe,ij})}+\frac{\Delta p_{ij}^{T}\Delta v_{ij}}{||\Delta p_{ij}\left|\right|}\geq 0$$
  This constitutes a safety function with a relative degree of r=1; therefore, the ZCBF theory is employed for the proof.
  According to Theorem 1, to guarantee the forward invariance of the set defined by $h_{ij}\geq 0$, the controller $u_{i}$ must satisfy the condition ${\dot{h}}_{ij}+\lambda_{1}h_{ij}\geq 0$ (choosing the class K function as $\alpha \left(h\right)=\lambda_{1}h$). In Equation (41), it was derived that this condition is equivalent to $L_{f}h_{ij}+L_{g}h_{ij}\left(u_{i}-u_{j}\right)+\lambda_{1}h_{ij}\geq 0$. Through the symmetric allocation strategy in Equation (42), the constraint on $u_{i}$ was formulated as $A_{ij}u_{i}\geq b_{ij}\left(x\right)$. The feasible set K(x) of the QP controller $u_{i}^{*}$ in Equation (51) is defined such that it must satisfy Equation (43). Consequently, the optimal solution $u_{i}^{*}$ necessarily satisfies the ZCBF condition. By Theorem 1, $u_{i}^{*}$ guarantees that $C_{agent}$ is forward-invariant.
(b) Forward Invariance of $C_{vel}$ (Velocity Limit):
  The set $C_{vel}$ is characterized by the safety function $h_{v,i}\left(x\right)\geq 0$, defined as
  $$h_{v,i}\left(v\right)=v_{max}^{2}-{\left|\right|v_{i}||}^{2}\geq 0$$
  This is similarly a safety function with a relative degree of r=1, necessitating the use of ZCBF theory.
  To guarantee the forward invariance of $h_{v,i}\geq 0$, the controller $u_{i}$ must satisfy the condition ${\dot{h}}_{v,i}+\lambda_{3}h_{v,i}\geq 0$. In Equation (49), this condition was derived to be equivalent to $L_{f}h_{v,i}+L_{g}h_{v,i}u_{i}+\lambda_{3}h_{v,i}\geq 0$, which corresponds to the linear inequality $A_{v,i}u_{i}\leq b_{v,i}\left(x\right)$. The feasible set K(x) of the QP controller $u_{i}^{*}$ is similarly defined such that it must strictly satisfy Equation (50). Consequently, $u_{i}^{*}$ guarantees that $C_{vel}$ is forward-invariant.
(c) Forward Invariance of $C_{obs}$ (Obstacle Avoidance):
  The set $C_{obs}$ is characterized by the safety function $h_{io}\left(x\right)\geq 0$, where
  $$h_{io}\left(p\right)=\left|\right|p_{i}-p_{o}\left|\right|-d_{obs}\geq 0$$
  This function has a relative degree of r=2, necessitating the use of HOCBF theory (Definition 5).
  To guarantee the forward invariance of $h_{io}\geq 0$, we define $\psi_{0}=h_{io}$ and $\psi_{1}={\dot{\psi }}_{0}+k_{1}\psi_{0}$ and require the controller $u_{i}$ to satisfy
  $$L_{f}\psi_{1}+L_{g}\psi_{1}u_{i}+k_{2}\psi_{1}\geq 0$$
  In Equation (46), it was proven that this HOCBF condition is equivalent to the QP constraint $A_{io}u_{i}\geq b_{io}\left(x\right)$. The feasible set K(x) of the QP controller $u_{i}^{*}$ is defined such that it must satisfy Equation (47). Consequently, $u_{i}^{*}$ guarantees that $C_{obs}$ is forward-invariant.

In summary, the QP controller $u_{i}^{*}$ is formulated to optimize the control input subject to the safety constraints characterizing $C_{agent}$, $C_{obs}$, and $C_{vel}$. Consequently, under the condition that the hard constraints are feasible (i.e., the optimal slack variable satisfies $\delta^{*}=0$), the control input strictly satisfies all barrier function inequalities, thereby guaranteeing that the intersection set $C_{safe}$ is forward-invariant. In extreme scenarios where $\delta^{*}>0$, the strictly forward-invariant property is locally relaxed, and the controller operates to minimize the violation of the safety boundaries to maintain system solvability and operational continuity.

The proof is complete. □

### 4.2. Convergence Analysis

In Section 4.1, it was proven that the system remains safe under the effect of the QP controller $u_{i}^{*}$, meaning that $C_{safe}$ is forward-invariant. Building on this, this section further analyzes the system’s convergence, proving that while maintaining safety, the system converges to the desired coverage objective (i.e., $R_{i}\to R_{j}$).

LaSalle’s Invariance Principle [35] is employed to analyze system convergence. The Control Lyapunov Function $V_{clf}\left(x\right)$ defined in Section 3.1 is selected as the Lyapunov candidate function for this analysis:

(51) $$V_{clf}\left(x\right)=V_{c}\left(p\right)+V_{k}\left(v\right)=\sum_{i=1}^{N}\frac{T_{i}{\left(p\right)}^{2}}{E_{i}}+\frac{1}{2}\sum_{i=1}^{N}v_{i}^{T}v_{i}.$$

According to Theorem 3, the system trajectory x(t) is confined within a compact set $\Omega =\{x\in C_{safe}|V_{clf}\left(x\right)\leq V_{clf}\left(x\left(0\right)\right)\}$, and $V_{clf}\left(x\right)\geq 0$ holds true.

**Theorem 4.**  *(System Convergence) Under the conditions of Theorem 3, and employing the random perturbation mechanism (Remark 2) to escape non-optimal local minima (deadlocks), the closed-loop system formed by the QP controller $u_{i}^{*}$ in Equation (51) will converge to the largest invariant set M. This set M is contained within the set S defined as*

(52) $$S=\{x\in \Omega |v_{i}=0,\forall i\in N\}.$$

*This implies that the system will ultimately converge to a static state where all AUV velocities are zero.*

**Proof of Theorem 4.**  To apply LaSalle’s Principle, first compute the time derivative ${\dot{V}}_{clf}$ of the Lyapunov function (52) under the effect of the actual QP controller $u_{i}^{*}$. Based on the derivation in Equation (27), we have

(53) $${\dot{V}}_{clf}=\sum_{i=1}^{N}v_{i}^{T}\left(\nabla_{p_{i}}V_{c}+u_{i}^{*}\right).$$

To simplify the derivation, following the settings in Equations (28) and (29), let the proportional gain of the nominal controller be $k_{p}=1$.

Express the actual controller $u_{i}^{*}$ as $u_{i}^{*}={\hat{u}}_{i}+\left(u_{i}^{*}-{\hat{u}}_{i}\right)$, where $\delta_{u}=u_{i}^{*}-{\hat{u}}_{i}$ represents the correction term generated by the QP to satisfy safety constraints. Substituting this into (54),

(54) $${\dot{V}}_{clf}=\sum_{i=1}^{N}v_{i}^{T}\left(\nabla_{p_{i}}V_{c}+{\hat{u}}_{i}+\left(u_{i}^{*}-{\hat{u}}_{i}\right)\right).$$

Now, substitute the nominal controller ${\hat{u}}_{i}=-\nabla_{p_{i}}V_{c}-k_{d}v_{i}$ into Equation (55):

(55) $${\dot{V}}_{clf}=\sum_{i=1}^{N}v_{i}^{T}\left(\nabla_{p_{i}}V_{c}+\left(-\nabla_{p_{i}}V_{c}-k_{d}v_{i}\right)+\left(u_{i}^{*}-{\hat{u}}_{i}\right)\right).$$

Simplifying yields

(56) $${\dot{V}}_{clf}=\sum_{i=1}^{N}v_{i}^{T}\left(-k_{d}v_{i}+\left(u_{i}^{*}-{\hat{u}}_{i}\right)\right)=\underset{NominalDamping}{\underset{︸}{-k_{d}\sum_{i=1}^{N}{\left||v_{i}|\right|}^{2}}}+\underset{\mathrm{CBF}Perturbation}{\underset{︸}{\sum_{i=1}^{N}v_{i}^{T}\left(u_{i}^{*}-{\hat{u}}_{i}\right)}}.$$

Equation (57) indicates that ${\dot{V}}_{clf}$ is composed of two parts. As long as $v_{i}\neq 0$, the first term is negative semi-definite, representing the desired damping term. The second term is the CBF perturbation term, whose sign is indefinite. When safety constraints are active (i.e., $u_{i}^{*}\neq {\hat{u}}_{i}$), these constraints may compromise the descent of the CLF, potentially causing ${\dot{V}}_{clf}$ to be temporarily non-negative.

Although ${\dot{V}}_{clf}$ is not a strictly negative semi-definite function, since the system trajectory x(t) is proven to be confined within the compact set Ω, LaSalle’s Invariance Principle dictates that the system trajectory x(t) must converge to the largest invariant set *M* contained within Ω. This set *M* is a subset of the set *S* where ${\dot{V}}_{clf}=0$.

(57) $$S=\left\{x\in \Omega |{\dot{V}}_{clf}=0\right\}=\{x\in \Omega |-k_{d}\sum ||v_{i}|{|}^{2}+\sum v_{i}^{T}\left(u_{i}^{*}-{\hat{u}}_{i}\right)=0\}.$$

If $v_{i}=0$ (for all *i*), then ${\dot{V}}_{clf}=0+0=0$. Consequently, all static states where the system velocities are zero are included in the set *S*.

(58) $$\{x\in \Omega |v_{i}=0,\forall i\}\subseteq S.$$

The largest invariant set *M* to which the system converges must satisfy M⊆S. According to the definition of an invariant set, any trajectory starting from within *M* must remain in *M* forever. For a trajectory to remain in *M* permanently, it must consistently satisfy ${\dot{V}}_{clf}=0$. Based on the analysis of Equation (59), this requires that the trajectory must satisfy $v_{i}\left(t\right)\equiv 0$ identically.

If $v_{i}\left(t\right)\equiv 0$ holds identically, then its derivative ${\dot{v}}_{i}\left(t\right)=0$ must also hold. According to the system dynamics in Equation (10), where ${\dot{v}}_{i}=u_{i}^{*}$, this implies that all states within the largest invariant set *M* must satisfy

(59) $$M\subseteq \{x\in \Omega |v_{i}=0,{\dot{v}}_{i}=u_{i}^{*}=0,\forall i\}.$$

The proof is complete. □

The static equilibrium point to which the system converges must satisfy $v_{i}=0$ and $u_{i}^{*}=0$. We now analyze the condition under which $u_{i}^{*}=0$. When $v_{i}=0$, the nominal controller simplifies to ${\hat{u}}_{i}=-k_{p}\nabla_{p_{i}}V_{c}$. Consequently, the QP problem transforms into

(60) $$u_{i}^{*}=\arg\min_{u_{i}\in K\left(x{|}_{v=0}\right)}\left|\right|u_{i}-\left(-k_{p}\nabla_{p_{i}}V_{c}\right)|{|}^{2},$$

where $K(x{|}_{v=0})$ denotes the safe feasible set at the instant when $v_{i}=0$. The condition $u_{i}^{*}=0$ being the solution to this QP occurs in two scenarios:

(a) Ideal Equilibrium: In this case, the safety constraints are inactive or the nominal control naturally satisfies them. The system stops because the coverage objective is achieved, i.e., $\nabla_{p_{i}}V_{c}=0$, leading to ${\hat{u}}_{i}=0$. This corresponds to the global or local minimum of the coverage cost function where workload consensus is reached.
(b) Constrained Equilibrium (Deadlock): In this case, the safety constraints are active. The system stops not because the task is complete ($\nabla_{p_{i}}V_{c}\neq 0$) but because the nominal control input ${\hat{u}}_{i}$ is exactly strictly opposed by the safety correction term required to maintain the forward invariance of $C_{safe}$. Mathematically, this occurs when the negative gradient direction aligns perfectly with the normal vector of the safety boundary, creating a local minimum where $u_{i}^{*}=0$ despite ${\hat{u}}_{i}\neq 0$.

With the activation of the perturbation mechanism described in Remark 2, the system is capable of escaping these unstable equilibria. Consequently, the system will ultimately converge to the ideal equilibrium (Case a), where the following conditions are satisfied:

(61) $$v_{i}=0\;\mathrm{and}\;\nabla_{p_{i}}V_{c}=0,\;\forall i\in N.$$

According to Lemma 2, $\nabla_{p_{i}}V_{c}=0$ is the necessary condition for the coverage cost function $V_{c}\left(p\right)=\sum \frac{T_{i}{\left(p\right)}^{2}}{E_{i}}$ to attain a (local) minimum. This condition is satisfied if and only if the workload-to-capability ratios $R_{i}\left(p\right)=T_{i}/E_{i}$ achieve consensus across all AUVs. Consequently, the system is proven to converge to a static state with zero velocity, simultaneously realizing the optimal coverage state characterized by heterogeneous task workload balance.

**Remark 2.**  *While (Case b) represents a theoretical local minimum (deadlock) inherent to gradient-based methods in cluttered environments, it is often an unstable equilibrium. To ensure the system converges to the Ideal Equilibrium (Case a), a practical random perturbation mechanism is implemented. If the system detects a static state ($\left|\right|v_{i}\left|\right|<\epsilon_{v}$) while the coverage gradient is non-zero ($\left|\right|\nabla_{p_{i}}V_{c}\left|\right|>\epsilon_{g}$), a small random perturbation vector $\delta_{rand}$ is temporarily added to the nominal controller ${\hat{u}}_{i}$. This breaks the symmetry of the force balance, allowing the agent to slide along the safety boundary and escape the deadlock.*

## 5. Simulation Experiments and Analysis

Numerical simulations were conducted to verify the effectiveness of the heterogeneous AUV cooperative coverage control framework proposed in this paper, which integrates CBFs with consensus theory. The simulation experiments were performed on a device equipped with an Intel Core i7-8750H processor (2.6 GHz), running the Windows 11 64-bit operating system. The algorithms were implemented using MATLAB R2023a. Two sets of comparative experiments were designed to separately verify the two core performance indicators of the control framework:

(a) Coverage Performance Verification: In an obstacle-free environment, this experiment validates whether the nominal controller can drive the heterogeneous AUV swarm to achieve consensus convergence of the task workload-to-capability ratio ($R_{i}\to R_{j}$).
(b) Safety Verification: In an environment containing static obstacles, this experiment validates whether the CBF-QP-based controller can strictly satisfy collision avoidance and state constraints while performing the coverage mission, thereby ensuring system safety.

The simulation mission area D is defined as a 5000 m × 5000 m two-dimensional rectangular sea region. The information density function (task weight) within the region is modeled as a Mixture of Gaussians [37] to simulate the distribution of regions of interest in a vast underwater environment:

$$\begin{array}{c} \phi \left(q\right)=\exp\left(-10^{-5}∥q-{\left[2500,4000\right]}^{T}{∥}^{2}\right)+\exp\left(-10^{-5}∥q-{\left[1200,1200\right]}^{T}{∥}^{2}\right)\\ +\exp(-10^{-5}∥q-{\left[3800,1200\right]}^{T}{∥}^{2})+0.3 \end{array}$$

This density function exhibits three high-value targets located at (2500,4000), (1200,1200), and (3800,1200).

Consider a swarm composed of N=5 heterogeneous AUVs, whose initial positions are randomly distributed within an error range of (−300,300) around the region’s center coordinates (2500,2500). To reflect heterogeneity, the comprehensive capability parameters $E_{i}$ are set to be different for each AUV. The sensing radius $R_{s}$ is set to 600 m, and the AUV safety distance is set to 50 m. Specific parameters and controller gains are listed in Table 1 below.

The core optimization QP problem (50) was solved using the MATLAB quadprog solver with the interior-point-convex algorithm. To ensure numerical stability and strict reproducibility, the discrete control update interval was set to Δt=0.1 s, and the spatial domain was discretized with a grid resolution of 1×1 m for Voronoi mass computation. Sensing radius $R_{s}=600$ m, safety threshold $d_{safe}=50$ m, and constraint activation hysteresis buffer $\epsilon_{hys}=0.5$ m. Furthermore, the random number generator seed was fixed (rng(1)) to guarantee consistent initialization of agent positions and perturbation logic across trials.

### 5.1. Experiment 1: Heterogeneous Cooperative Coverage in Obstacle-Free Environment

**Figure 2:**
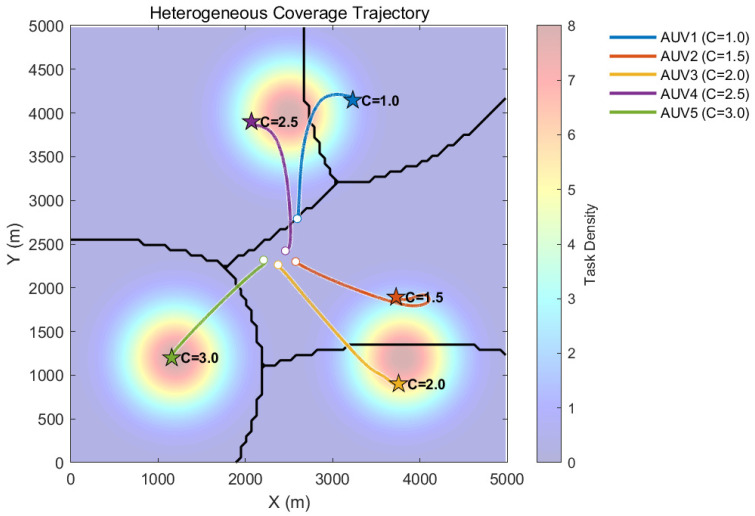
Cooperative coverage trajectories and spatial distribution of a heterogeneous AUV swarm in an obstacle-free environment.

**Figure 3:**
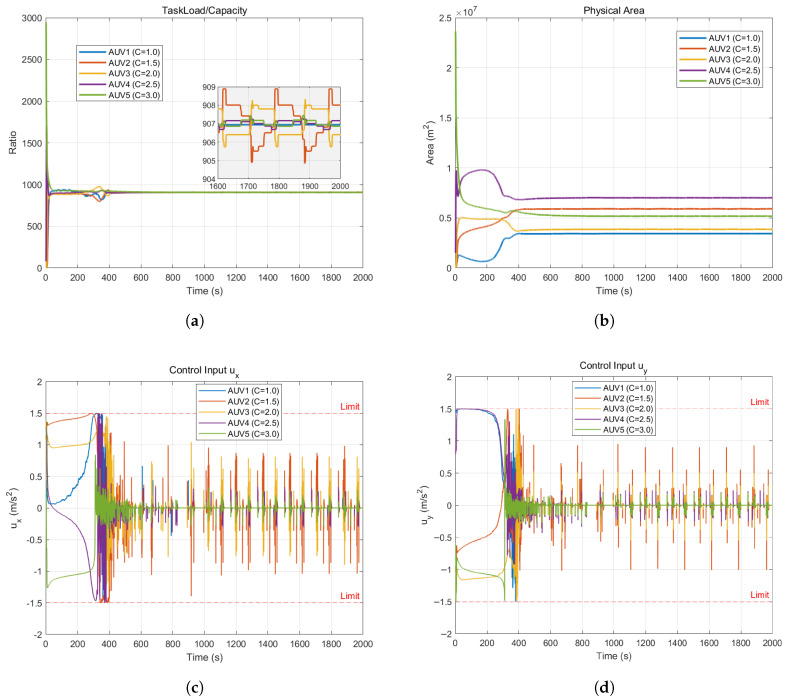
Coverage control state in an obstacle-free environment. (**a**) The taskload-to-capability ratio. (**b**) Spatial area allocation. (**c**) Control input $u_{x}$. (**d**) Control input $u_{y}$.

The experimental results of heterogeneous cooperative coverage in an obstacle-free environment are shown in Figure 2 and Figure 3.

Figure 2 illustrates the optimal coverage trajectories and the final Voronoi partition of the heterogeneous AUV swarm in an obstacle-free environment. Upon initiation of the simulation, the five AUVs rapidly disperse from their initial clustered state towards the three high-intensity Gaussian hotspots distributed within the mission area. Driven by the nominal controller, the AUVs automatically allocate target regions according to their individual capabilities: The stronger agents, AUV 5 and AUV 4, do not congregate at the same hotspot. Instead, they maneuver towards the high-density core regions located in the bottom-left and top areas, respectively, thereby occupying the primary task peaks. Conversely, the weaker AUV 1 is assigned to the peripheral region of the top hotspot. The black Voronoi partition lines in the figure clearly demonstrate the result of the capability-based weighted partition. The partition boundaries are not simple geometric perpendicular bisectors; instead, they are curved towards the weaker side. This curvature allows high-capability AUVs to govern broader high-value regions, providing intuitive validation of the algorithm’s adaptability in spatial allocation based on heterogeneity.

Figure 3a illustrates the dynamic evolution of the core performance metric—the task workload-to-capability ratio. The system begins in a state of extreme imbalance due to the random and clustered initial positions of the AUVs, evidenced by drastic disparities in load ratios ranging from near 0 to over 2500. However, as the control algorithm intervenes, the adaptive weighting mechanism drives the curves to rapidly converge, reaching a unified steady-state value of approximately 908 around t=400 s. While the zoomed-in view reveals minor periodic fluctuations attributed to the dynamic adjustment of Voronoi boundaries on the discrete simulation grid, these deviations are negligible and strictly confined near the convergence value. This result provides robust evidence that the algorithm successfully achieves the consensus equilibrium objective, ensuring that the actual workload undertaken by each AUV is strictly proportional to its physical capability.

Figure 3b illustrates the temporal evolution of the physical area occupied by each AUV, corroborating the impact of system heterogeneity. In contrast to the uniform convergence observed in workload ratios, the physical area trajectories exhibit a distinct stratified distribution at steady state, with AUV 4 occupying the largest area (approximately 0.7 × 10^7^ $m^{2}$) and the least capable agent, AUV 1, occupying the smallest (approximately 0.35 × $10^{7}$ m^2^). Notably, the physical area is not strictly proportional to capability magnitude—exemplified by the most capable agent, AUV 5, occupying a slightly smaller area than AUV 4—because spatial allocation is heavily influenced by environmental information density; AUV 5 secures a high-density core region where a smaller physical footprint suffices to accumulate a substantial workload, whereas AUV 4 covers a larger portion of the low-density periphery. This phenomenon underscores the algorithm’s ability to synergistically balance capability heterogeneity and environmental distribution to achieve an optimal spatial allocation.

Figure 3c,d depict the temporal evolution of the control inputs for the AUV swarm. During the initial phase (0–300 s), the control inputs are predominantly saturated, adhering tightly to the physical constraint boundaries of $\pm 1.5\;{\mathrm{m/s}}^{2}$ (indicated by red dashed lines) as the AUVs traverse significant distances; this demonstrates the algorithm’s capacity to fully exploit the AUVs’ maximum maneuvering capabilities to minimize convergence time. Upon approaching the target regions, the control magnitudes rapidly decrease and stabilize, though some high-frequency switching is observed in the steady state—an artifact attributed to minor discrete jumps of Voronoi centroids at grid edges and the continuous fine-tuning required for strict workload balancing. Crucially, all control commands remain strictly within the predefined hard constraints throughout the entire process, thereby verifying the controller’s safety and feasibility regarding dynamic compliance.

As observed in Figure 3c,d, high-frequency oscillations occur when the AUVs operate near safety boundaries. This switching behavior is characteristic of QP-based controllers strictly enforcing hard constraints in a discrete-time setting. While mathematically ensuring forward invariance, such chattering is undesirable for physical marine actuators due to potential mechanical wear and energy inefficiency. For practical implementation, this issue can be mitigated through several strategies:

(a) Signal Smoothing: Applying a low-pass filter to the QP-generated acceleration command $u_{i}^{*}$ before it reaches the thruster drivers.
(b) Relaxed Barrier Functions: Implementing a boundary layer or using relaxed class-K functions in the CBF formulation to allow for smoother transitions between safe and unsafe sets.
(c) Input Regularization: Increasing the regularization weight in the QP cost function to penalize rapid changes in control effort (${∥\Delta u∥}^{2}$).

The results from Experiment 1 demonstrate that, in an obstacle-free environment, the proposed control framework effectively accommodates the heterogeneous characteristics of the AUV swarm. By leveraging the adaptive adjustment of Voronoi weights, the algorithm successfully steers AUVs toward task regions commensurate with their respective capabilities, thereby realizing a global consensus on the workload-to-capability ratio and validating the effectiveness of the nominal controller.

### 5.2. Experiment 2: Safe Coverage Control in Obstacle Environments

**Figure 4:**
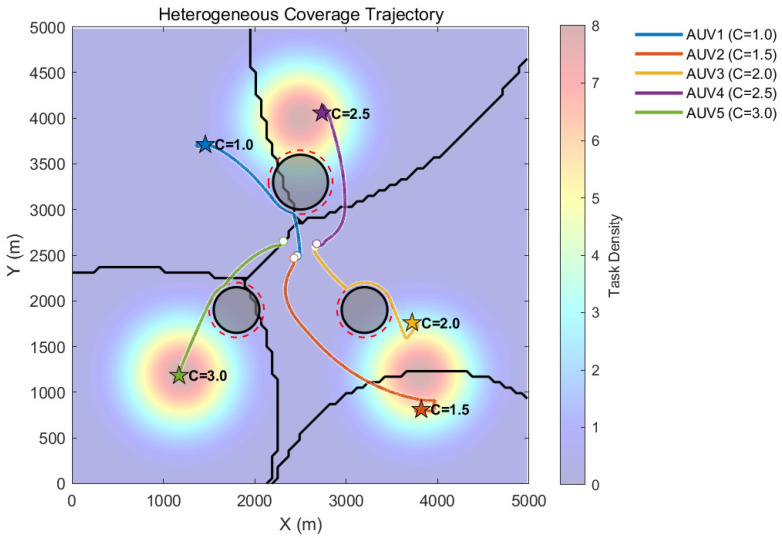
Cooperative coverage trajectories and spatial distribution of a heterogeneous AUV swarm in an obstacle environment.

**Figure 5:**
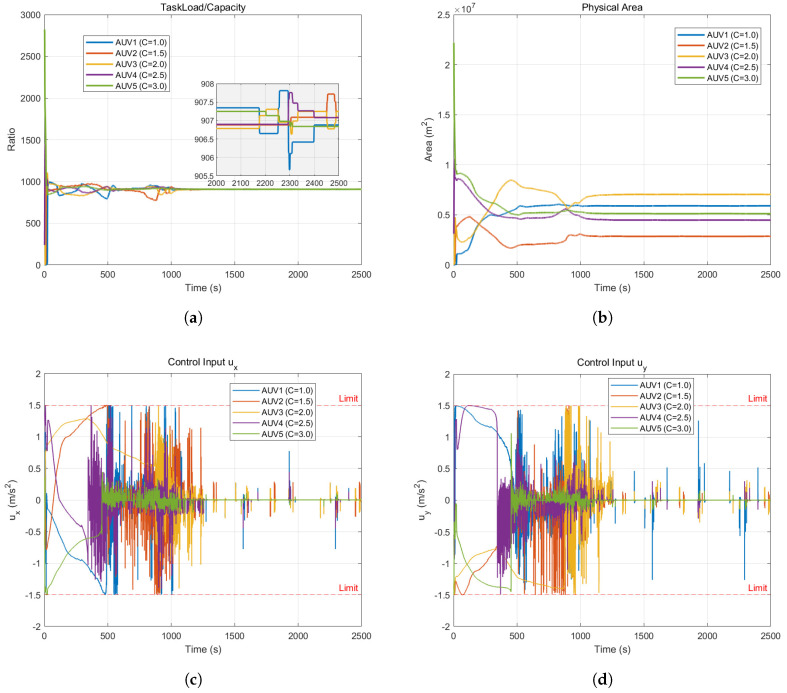
Coverage control state in an obstacle environment. (**a**) The taskload-to-capability ratio. (**b**) Spatial area allocation. (**c**) Control input $u_{x}$. (**d**) Control input $u_{y}$.

**Figure 6:**
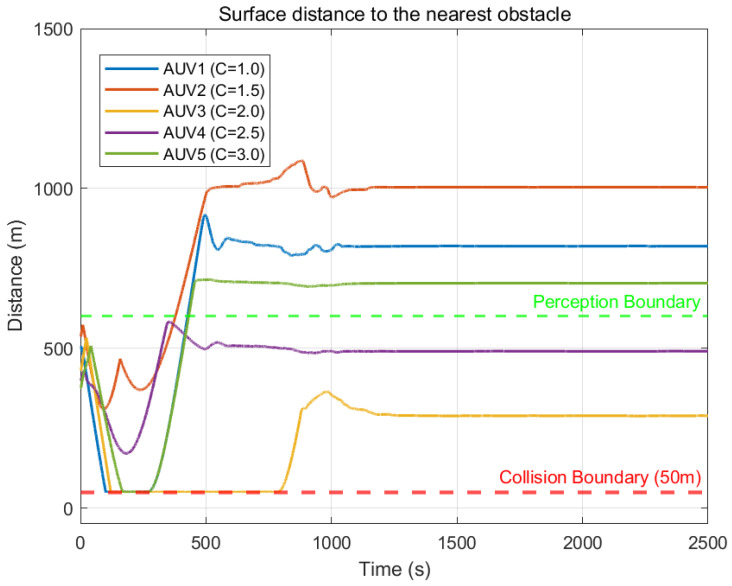
Distance between AUV and nearest obstacle.

To evaluate the obstacle avoidance capability, three circular obstacles were established within the simulation area at coordinates (2500,3300), (1800,1900), and (3200,1900), with radii of 300 m, 250 m, and 250 m, respectively. The experimental results regarding safe coverage control in this obstacle-laden environment are presented in Figure 4, Figure 5 and Figure 6.

Figure 4 visually demonstrates the path planning and spatial allocation capabilities of the heterogeneous AUV swarm in the presence of static obstacles. Observing the trajectories, the five AUVs depart from their initial clustered region and accurately identify the gray circular obstacles located along their paths toward the three Gaussian target hotspots. Unlike the linear trajectories observed in the obstacle-free environment, the trajectories in this experiment exhibit smooth curvilinear circumvention characteristics when approaching obstacles. Ultimately, the AUVs not only safely bypass the obstacles but also form a rational Voronoi coverage configuration based on their respective capability weights $E_{i}$. The Voronoi partition lines exhibit adaptive curvature in response to the environmental geometric constraints, verifying the effectiveness of the heterogeneous planning algorithm in complex topological environments.

Figure 5a reflects the cooperative performance and robustness of the swarm under perturbed conditions. Compared to the obstacle-free environment, the workload ratio curves in this experiment exhibit more significant fluctuations during the convergence process, particularly between t=200 s and 800 s. This is attributed to the presence of obstacles, which temporarily obstruct the sensing line-of-sight of certain AUVs or alter the Voronoi neighborhood topology, leading to sudden jumps in local workload calculations. Nevertheless, as the AUVs circumnavigate the obstacles and enter the target regions, the adaptive weight update law rapidly eliminates these disturbance-induced biases. Ultimately, all curves stably converge to a unified constant value after t=1000 s, demonstrating that the proposed consensus algorithm possesses effective disturbance rejection capabilities.

The stratification phenomenon in the curves of Figure 5b clearly embodies the heterogeneous characteristics: At steady state, AUVs with higher capabilities tend to occupy larger physical areas than those with lower capabilities. Notably, during the obstacle avoidance phase around t=400 s, some curves display sharp spikes or dips. This occurs because the AUVs are forced to temporarily compress or stretch their Voronoi cells to avoid obstacles. This dynamic area adjustment mechanism proves that the algorithm can flexibly manage the conflict between geometric spatial constraints and mission workload requirements, effectively trading temporary spatial optimality for system safety and eventual workload balance.

Figure 5c,d depict the time-domain evolution of acceleration control inputs. Following an initial phase of saturation at the $\pm 1.5\;{\mathrm{m/s}}^{2}$ boundaries to maximize speed, the intermediate obstacle avoidance phase features dense high-frequency switching, representing the real-time interplay between the nominal controller (target seeking) and safety constraints (obstacle avoidance).

Figure 6 displays the real-time Euclidean distance between each AUV and the nearest obstacle surface, with the red dashed line indicating the mandatory 50 m collision boundary. Simulation results show that during the avoidance period (t=100 s to 500 s), while distance curves for AUV 4 and AUV 3 drop sharply as they approach obstacles, the minima strictly remain above the red dashed line. This confirms that the HOCBF-based safety controller effectively intervenes upon detecting collision risks to generate sufficient repulsive acceleration, ensuring the system state remains within the safety set for a rigorous zero-collision guarantee.

The results of Experiment 2 demonstrate the robustness of the proposed control framework. Facing complex conditions with multiple static obstacles, the algorithm not only strictly enforces safe collision avoidance distances but also successfully guides the heterogeneous swarm to overcome disturbances caused by environmental topology changes, ultimately achieving efficient and balanced cooperative coverage.

### 5.3. Experiment 3: Comparative Experiments and Quantitative Analysis

To comprehensively evaluate the performance of the proposed hierarchical control framework, comparative experiments were conducted against three baseline methods under the same environmental conditions as Experiment 2 (containing multiple static obstacles). The simulation duration was set to 1500 s to ensure sufficient time for convergence. The baseline strategies are defined as follows:

(a) Standard Lloyd’s Algorithm: A classic coverage control method based on Voronoi partitioning that drives agents toward centroids without explicit obstacle avoidance constraints.
(b) APF-Based Method: A method combining the nominal coverage controller with Artificial Potential Fields to handle obstacle avoidance reactively.
(c) CBF-Only Method: A simplified strategy that applies the proposed safety constraints but utilizes a standard position-based nominal controller without the heterogeneous workload-to-capability consensus mechanism.

The quantitative results are summarized in Table 2. The performance metrics include

(a) Coverage Cost (CCost): Defined as the standard deviation of the workload-to-capability ratio ($R_{i}$) across the swarm. A lower $V_{c}$ indicates a more balanced task allocation aligned with agent heterogeneity.
(b) Coverage Time (CTime): Time required to reach the steady-state coverage configuration.
(c) Min Obs Dist (MOD): The minimum Euclidean distance recorded between AUV and the nearest obstacle during the entire simulation (safety threshold $d_{safe}=50$ m).
(d) Min AUV Dist (MAD): The minimum Euclidean distance recorded between any AUV during the entire simulation.
(e) Violations (Vio.): Total accumulated count of time steps where safety constraints were violated (i.e., distance < 50 m).
(f) Runtime: Average computation time per control step.

**Figure 7:**
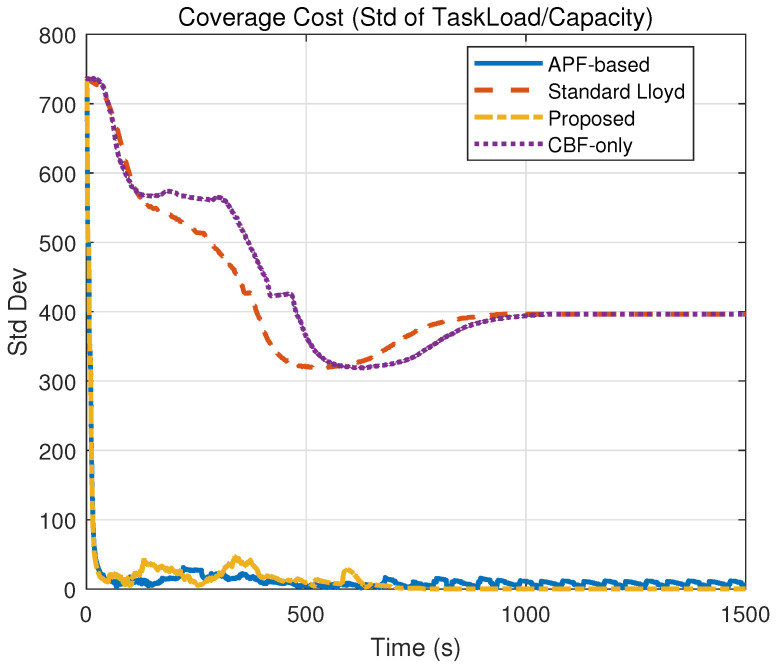
Comparison of coverage cost evolution.

The evolution of coverage performance, quantified by the coverage cost in Table 2 and visualized in Figure 7, demonstrates the superior efficiency of the proposed framework. As indicated by the rapidly descending yellow curve in Figure 7, the proposed method achieves a steady-state cost of 0.15 within 702 s, signifying a heterogeneous load balance where the swarm reaches the global optimum efficiently. In contrast, the Standard Lloyd and CBF-only methods fail to converge, maintaining a high cost of 396.41 due to unresolved conflicts, while the APF-based method settles at a suboptimal local minimum of 5.85 after a convergence process.

**Figure 8:**
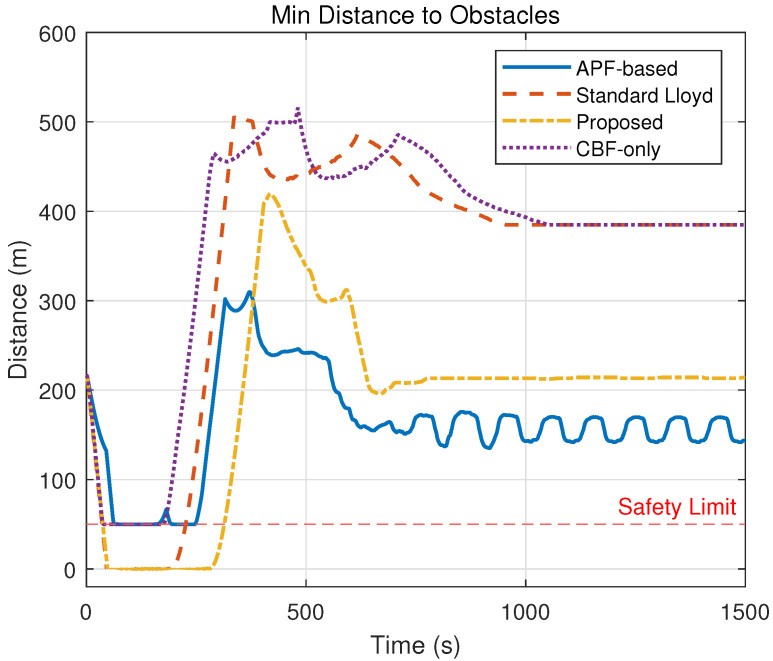
Minimum distance to static obstacles.

**Figure 9:**
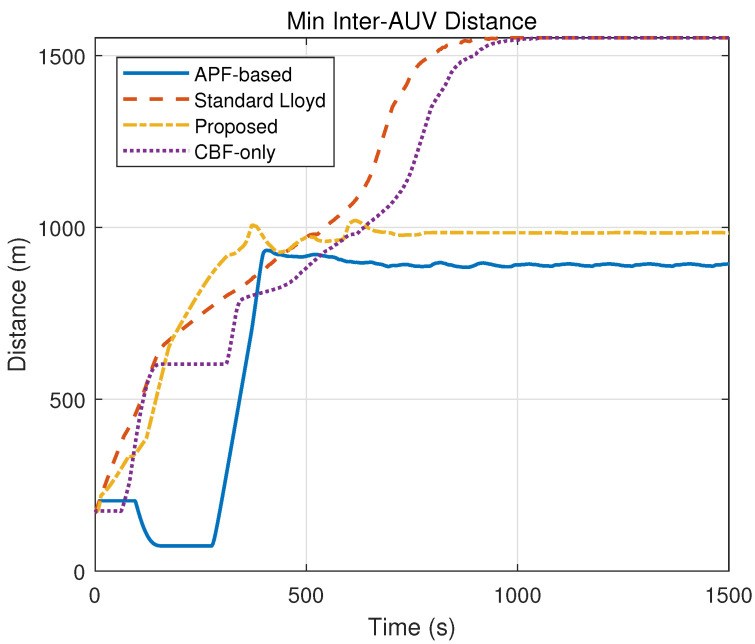
Minimum inter-agent distance.

Safety performance is validated through the obstacle distance metrics in Table 2 and the time-domain evolution in Figure 8 and Figure 9. Specifically, the Standard Lloyd and APF-based strategies exhibit failures, with their distance curves in Figure 8 frequently dropping to 0.00 m (crossing the 50 m safety threshold), which corresponds to the massive violation counts of 4252 and 6978, respectively. Conversely, the proposed method strictly maintains a minimum distance of 50.02 m with 0 violations, and as shown in Figure 9, it maintains a stable inter-agent separation, proving that the hierarchical ZCBF/HOCBF constraints effectively enforce hard safety boundaries.

**Figure 10:**
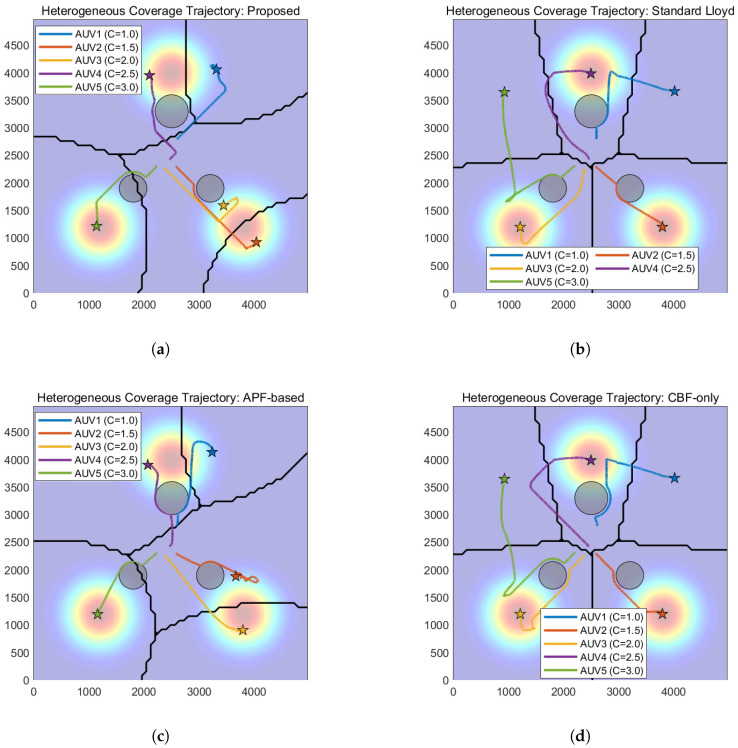
Spatial trajectory comparison. (**a**) The proposed method. (**b**) Lloyd’s method. (**c**) APF-based method. (**d**) CBF-only method.

The spatial trajectories presented in Figure 10, combined with the safety violation statistics in Table 2, highlight the critical role of the consensus mechanism in preventing deadlocks. The proposed method generates paths (Figure 10a) that successfully navigate obstacles. Meanwhile, the collision-prone paths of the Lloyd method (Figure 10b) and the oscillating paths of the APF method (Figure 10c) further confirm their unsuitability for complex environments compared to the feasible solution found by this paper.

**Figure 11:**
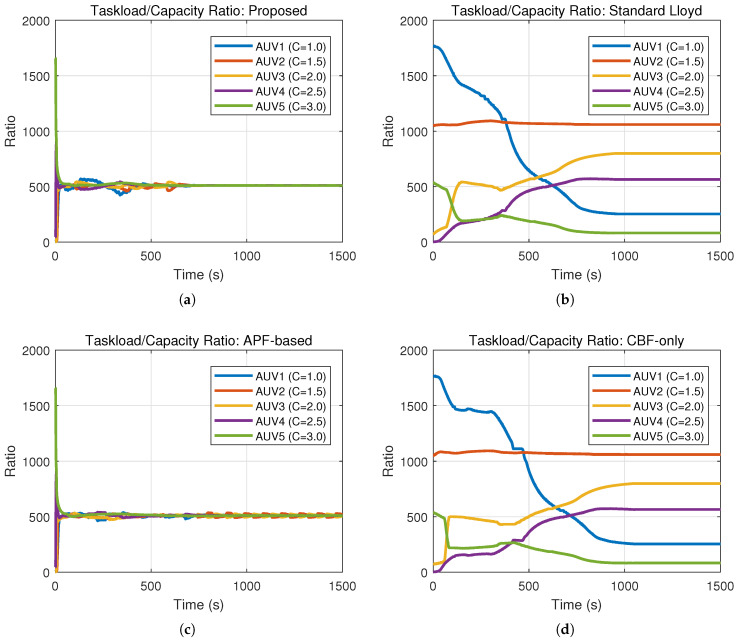
Taskload-to-capability ratio ($R_{i}$) evolution. (**a**) The proposed method. (**b**) Lloyd’s method. (**c**) APF-based method. (**d**) CBF-only method.

Figure 11 provides visual confirmation of the heterogeneous load balancing capability, which is mathematically represented by the low cost standard deviation of $V_{c}=0.15$. In the proposed method (Figure 11a), the workload-to-capability ratio curves for all five heterogeneous AUVs converge to a single consensus value, demonstrating that the task load is allocated proportional to each AUV’s capability $E_{i}$. This contrasts with the divergent or chaotic curves observed in the baseline methods (Figure 11b–d)—such as the Standard Lloyd case where capable AUVs remain underutilized while weaker ones are overloaded—thereby validating that the proposed consensus-based nominal controller effectively exploits system heterogeneity to optimize global coverage.

## 6. Conclusions

This paper addresses the cooperative coverage control problem for heterogeneous AUV swarms in complex underwater environments by proposing a hierarchical control framework based on QP that integrates CBFs with consensus theory. At the performance layer, a nominal controller ${\hat{u}}_{i}$ is designed leveraging CLF concepts and consensus theory to minimize the cost function $V_{c}=\sum \left(T_{i}^{2}/E_{i}\right)$, thereby driving the workload-to-capability ratio $R_{i}$ toward global consensus. At the safety layer, ZCBFs and HOCBFs are employed to transform inter-AUV collision avoidance, obstacle avoidance, and velocity limits into linear constraints for the controller. Theoretical analysis confirms the forward invariance of the safe set and the convergence of the coverage mission, while simulation experiments validate that the swarm achieves efficient heterogeneous load balancing while prioritizing safety constraints via a soft-constraint formulation.

Regarding the gap between the theoretical model and practical deployment, future work will focus on integrating the proposed high-level planner with high-fidelity AUV dynamic models. Specifically, we will investigate the combination of this framework with robust low-level controllers to actively compensate for hydrodynamic damping and actuator saturation. Furthermore, incorporating braking-distance-aware safety margins into the QP formulation will be explored to enhance the system’s applicability in real-world ocean currents. We also plan to extend the collision avoidance capabilities to address dynamic non-cooperative obstacles by integrating state estimation and time-varying barrier functions into the CBF-QP optimization.

## Figures and Tables

**Table 1:** Simulation parameter settings.

Parameter Symbol	Physical Meaning	Value/Unit
*N*	Number of AUVs	5
*E*	Heterogeneous capability vector	[1.0, 1.5, 2.0, 2.5, 3.0]
$R_{s}$	Sensor sensing radius	600 m
$v_{max}$	Maximum speed limit	5 m/s
$u_{max}$	Maximum acceleration limit in X/Y direction	$1.5\;{\mathrm{m/s}}^{2}$
$d_{safe}^{AUV}$	AUV safety distance	50 m
$k_{p},k_{d}$	Nominal controller gain	0.5, 1.0
γ	CBF class function coefficient	0.5

**Table 2:** Quantitative performance comparison of different control strategies.

Method	CCost	CTime (s)	MOD (m)	MAD (m)	Vio.	Runtime (ms)
Proposed	0.15	702	50.02	170.45	0	0.71
Standard Lloyd	396.41	N/A	0.00	170.45	4252	0.20
APF-Based	5.85	730	0.00	72.67	6978	0.21
CBF-Only	396.41	N/A	50.01	170.45	0	0.73

## Data Availability

The original contributions presented in this study are included in the article. Further inquiries can be directed to the corresponding author.
